# Glial fibrillary acidic protein is pathologically modified in Alexander disease

**DOI:** 10.1016/j.jbc.2024.107402

**Published:** 2024-05-21

**Authors:** Ni-Hsuan Lin, Wan-Syuan Jian, Natasha Snider, Ming-Der Perng

**Affiliations:** 1 Institute of Molecular Medicine, National Tsing Hua University, Hsinchu, Taiwan; 2 Department of Cell Biology and Physiology, University of North Carolina, Chapel Hill, North Carolina, USA; 3 School of Medicine, College of Life Sciences and Medicine, National Tsing Hua University, Hsinchu, Taiwan

**Keywords:** intermediate filament, glial fibrillary acidic protein, Alexander disease, ubiquitination, aggregation

## Abstract

Here, we describe pathological events potentially involved in the disease pathogenesis of Alexander disease (AxD). This is a primary genetic disorder of astrocyte caused by dominant gain-of-function mutations in the gene coding for an intermediate filament protein glial fibrillary acidic protein (GFAP). Pathologically, this disease is characterized by the upregulation of GFAP and its accumulation as Rosenthal fibers. Although the genetic basis linking GFAP mutations with Alexander disease has been firmly established, the initiating events that promote GFAP accumulation and the role of Rosenthal fibers (RFs) in the disease process remain unknown. Here, we investigate the hypothesis that disease-associated mutations promote GFAP aggregation through aberrant posttranslational modifications. We found high molecular weight GFAP species in the RFs of AxD brains, indicating abnormal GFAP crosslinking as a prominent pathological feature of this disease. *In vitro* and cell-based studies demonstrate that cystine-generating mutations promote GFAP crosslinking by cysteine-dependent oxidation, resulting in defective GFAP assembly and decreased filament solubility. Moreover, we found GFAP was ubiquitinated in RFs of AxD patients and rodent models, supporting this modification as a critical factor linked to GFAP aggregation. Finally, we found that arginine could increase the solubility of aggregation-prone mutant GFAP by decreasing its ubiquitination and aggregation. Our study suggests a series of pathogenic events leading to AxD, involving interplay between GFAP aggregation and abnormal modifications by GFAP ubiquitination and oxidation. More important, our findings provide a basis for investigating new strategies to treat AxD by targeting abnormal GFAP modifications.

Intermediate filaments (IFs) are one of the three major cytoskeletal systems that provide a dynamic platform and a versatile scaffold for coordinating redox signaling and stress response. Unlike microfilaments and microtubules, which exhibit structural polarity that enables their cellular functions, IFs are apolar polymers with flexible structures that support the organization of the cytoarchitecture and regulate key signaling pathways that control cell growth, differentiation, and survival. In humans, more than 70 IF proteins are classified into six major types, each of which is characterized by unique structural features and diverse functional properties.

Glial fibrillary acidic protein (GFAP), a type III intermediate filament protein, is a canonical maker of mature astrocyte, and its elevated expression is a hallmark feature of reactive gliosis (1). Like other IF proteins, GFAP possesses a modular structure consisting of a highly conserved central rod domain (2), which flanked by more variable N-terminal head and C-terminal tail that are low-complexity domains containing intrinsically disordered regions. The centrally located α-helical rod domain contains about 310 amino acids featuring long-range heptad repeats of amphipathic residues, which mediates coiled-coil dimer formation and represents the major driving force sustaining self-assembly. In astrocytes, GFAP together with lesser amounts of other IF proteins, such as vimentin (3), nestin (4), and synemin (5), constitute the glial filaments. The GFAP-containing glial filaments give the astrocyte its distinct morphology, with multiple extension of fine processes that contact with other cell types in the central nervous system (CNS). Unlike most IF genes, however, *gfap* is alternatively spliced, producing multiple isoforms that differ mainly in their tail domains (6). The predominant isoform is GFAP-α, which has been the focus of most studies. In the human CNS, GFAP-α accounts for 90% of the total GFAP (7, 8), with other isoforms accounting for most of the rest.

The high abundance of GFAP and its specific expression in astrocytes suggest an important function for this IF protein, but what that function might be has been incompletely understood (9). Previous studies using animal models showed that while *GFAP* *gfap* KOs exhibited relatively normal phenotypes (10, 11, 12, 13, 14), transgenic mice engineered to overexpress normal human *gfap* exhibited a fatal phenotype with their astrocytes containing abundant protein aggregates (15) that were similar to the Rosenthal fibers (RFs) of Alexander disease (AxD). The unexpected finding that RFs can form as the result of a primary change in GFAP levels lead to the discovery that AxD is caused by mutations in the gene coding for GFAP (16).

AxD is a primary genetic disorder of astrocyte characterized by the upregulation of GFAP and reactive astrocyte response. Based on the lesion locations in the CNS, AxD is classified into two major subtypes. Type I patients are usually early onset with frontal dominance characterized by developmental delays, psychomotor retardation, and seizures, whereas type II patients have onsets at all ages with hindbrain pathologies (17). Pathologically, AxD is characterized by accumulation of GFAP aggregates in the form of RFs. These fibrous globs are ubiquitinated protein aggregates composed mainly of GFAP (18) and a host of other proteins, including synemin (19), plectin (20), the small stress proteins αB-crystallin (21) and HSP27 (22), TDP43 (23), cyclin D2 (24), and the polyubiquitin-binding protein p62 (25). Genetically, mutations in *gfap* are found in more than 95% of patients clinically diagnosed with AxD, and there are now more than 100 such mutations causally associated with this disease (26). Most of these mutations are typically missense nucleotide changes predicting single-amino-acid substitutions spread throughout the entire GFAP sequence. Almost all mutations identified so far are heterozygous coding mutations, which are genetically dominant and therefore the mutated proteins are expected to act in a gain-of-function manner. Consistent with this notion, no null mutations for *gfap* have ever been reported for patients with AxD. In approximately one-third of all published studies, patients have mutations at just four amino acids, R79, R88, R239, and R416, which are highly homologous to hot spots for mutations in other IFs (27). Although most of the AxD mutations are simple missense mutations, some are more complex. These include, for instance, 3- to 6-bp insertions or deletions that result in either in-frame gain or loss of a few amino acids (28). In rare cases, whole exons have been deleted due to splice site mutations causing exon skipping (29, 30), but in both instances, the coding sequence remained in-frame. However, frame-shift mutations that alter C-terminal tail domain of GFAP have also been reported (31, 32). The AxD-associated *gfap* mutations are presumed to exert their effects through GFAP-α, but recent studies showed that GFAP mutations cause AxD not through the predicted amino acid change but by altering the splicing of *gfap* pre-mRNA (33).

Like other IF proteins, GFAP is a target for various posttranslational modifications (PTMs) at multiple sites. Phosphorylation of GFAP at selective sites has been implicated in regulating GFAP assembly dynamics (34), which might be important in its turnover (35) and in the progression of mitosis (36). In particular, increased phosphorylation of serine 13 of GFAP has been associated with caspase six activation and GFAP cleavage in AxD patients with early-onset forms, independent of the mutation they carry (37). Citrullination at multiple arginine residues in GFAP through deimination is another modification occurring in various pathological conditions, including AxD (38). The citrullinated GFAP renders it an autoantigen for autoimmune disease and other pathological conditions such as ischemia reperfusion injury and retinal degeneration (39, 40). Other PTMs such as glycosylation and lipoxidation have also been found in the RF of AxD brains (41, 42), implicating oxidative stress may contribute to the aggregate formation. Notably, the only cysteine residue in human GFAP at position 294 that is highly conserved across all type III IF protein is susceptible to lipoxidation *in vitro* (43) and in cultured cells through prostaglandins (44), further suggesting that GFAP is susceptible to oxidative modification in response to oxidative stress. Among these modifications, ubiquitination has also been described for proteins present in the RFs of AxD (45), but whether GFAP itself carries this modification has not yet been determined.

GFAP aggregates in the form of RFs bear strong similarities to other IF-specific inclusions that represent hallmarks of various IF-associated diseases (46), including keratins in Mallory-Denk bodies of liver diseases (47), desmin in granulofilamentous materials of desmin-related myopathies (48), and neurofilaments in Lewy bodies of Parkinson’s disease (49). The disease-associated IF proteins share two common molecular features including pathologic aggregation and aberrant PTMs (50). However, it is not clear whether and how these modifications contribute to or are the consequence of pathological inclusion. Given that AxD mutations often involve substitutions of the WT residues by cysteine in approximately 16% of the reported AxD cases, the higher nucleophilicity of this residue could render mutant GFAP more susceptible to additional modifications. In this study, we carry out experiments to determine whether cystine-generating AxD mutations promote aggregation through aberrant modifications that impact GFAP assembly and functional properties.

## Results

### GFAP is pathological modified in the RFs of AxD

**Figure 1:**
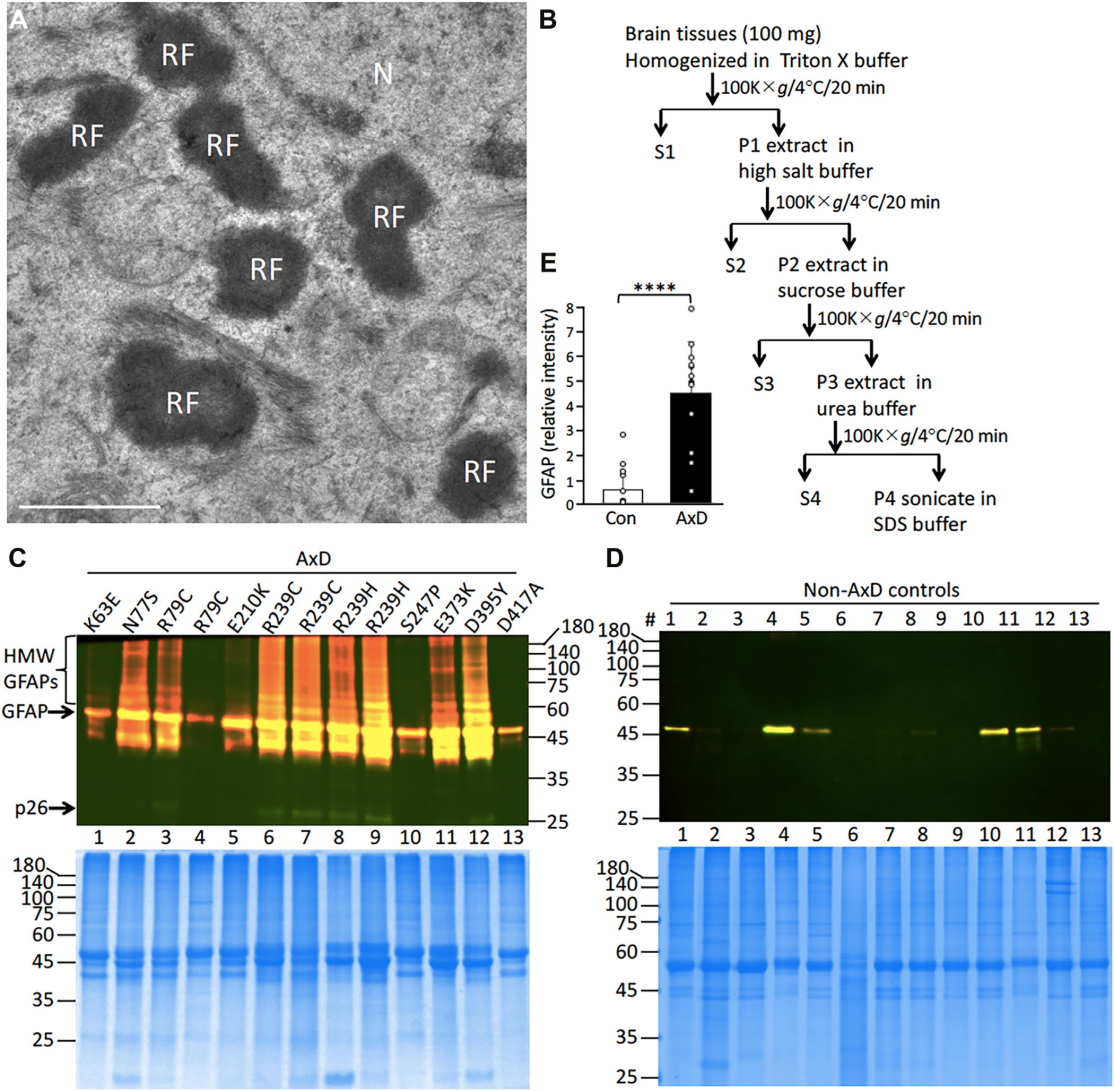
**Ultrastructural and biochemical analyses of Rosenthal fibers.***A*, EM showed electron-dense GFAP aggregates similar to Rosenthal fibers (RF) in the perinuclear region of the brain tissue from an AxD case carrying R239C GFAP mutation. N, nucleus. Bar represents 500 nm. *B*, schematic diagram of the biochemical extraction protocol used in this study. The RF-enriched fractions from AxD brain tissues (*C*, with GFAP mutations indicated on the *top*) and non-AxD controls (*D*, #1–13) were analyzed by immunoblotting using a monoclonal anti-GFAP antibody SMI-21 that recognized the N-terminal part of GFAP (*green* channel) and a polyclonal anti-GFAP antibody that recognized the C-terminal part of GFAP (*red* channel). Merged immunoblot showed the superimposition of both the *green* and *red* signals. Protein samples were loaded at 1 μg per lane and total protein profiles of each lane were visualized by Coomassie blue staining (*C* and *D*, *bottom* panel). GFAP and high molecular weight (HMW) species are indicated on the *right*. Molecular weight markers (in kDa) are indicated on either *left* or *right* side of the gel. *E*, quantification of GFAP levels in AxD samples compared to non-AxD controls. Data are mean ± SD. ∗∗∗∗*p* < 0.0001 (two-tailed *t* test). Each *white* dot represents a sample from a brain tissue (n = 13). GFAP, glial fibrillary acidic protein; p26, 26 kDa GFAP degradation product.

Given that AxD mutations usually caused GFAP aggregation, we asked whether GFAP could be aberrantly modified in a way that promotes GFAP aggregation. EM was initially performed to show the presence of GFAP aggregates in the form of RFs as electron-dense materials (Fig. 1*A*) in the brain tissue from an AxD patient carrying a R239C GFAP mutation. To analyze GFAP biochemically, RF-enriched fractions were prepared from the brain tissues of 13 AxD patients who carried different GFAP mutations (Table 1), along with 13 non-AxD controls. Human brain tissues were sequentially extracted by a fractionation protocol (Fig. 1*B*) that extracted soluble and filamentous GFAP into Triton X-soluble (Fig. S1) and urea-soluble (Fig. S2) fractions, while retaining urea-insoluble but SDS-soluble GFAP in the RF fraction. The RF fraction was analyzed using two anti-GFAP antibodies that we had characterized and validated in a previous study (51). Whereas the mouse anti-human GFAP monoclonal antibody recognized the rod domain of human GFAP spanning amino acids 179 to 206, the rabbit anti-panGFAP polyclonal antibodies were able to detect various forms of biochemically modified GFAP. Immunoblotting revealed a general increase of GFAP in AxD samples (Fig. 1*C*) compared to non-AxD controls (Fig. 1*D*). Coomassie blue–stained gels showed the total protein profiles in the samples analyzed, which had similar protein loading for each lane (Fig. 1, *C* and *D*, bottom panels). Although levels of full-length GFAPs were not consistently altered in AxD samples (Fig. 1*E*), a dramatic increase in high molecular weight (HMW) GFAP species were detected in six type I AxD cases (Fig. 1*C*, lanes 2, 6, 8, 9, 11, and 12), representing the youngest of the 13 patients analyzed. The early-onset type II AxD cases (Fig. 1*C*, lanes 3 and 7) also had detectable levels of higher forms of GFAP, which were not detected in the five late-onset cases (Fig. 1*C*, lanes 1, 4, 5, 10, and 13) and in any non-AxD controls (Fig. 1*D*). Notably, the levels of HMW GFAPs in AxD patients correlated with the detection of increased accumulation of GFAP degradation products, one of which was likely to be caspase 6-generated (Fig. 1*C*, p26 indicated by an arrow) (51).

**Table 1:** Clinical and genetic details of human postmortem tissue samples analyzed by immunoblotting

Case	GFAP mutation	Cause of death	Age of death		PMI	Sex	ID number
			Years	Days			
Control #1	-	Head injury	27	340	4 h	F	1711
Control #2	-	Drowning	2	0	9 h	M	5941
Control #3	-	Asphyxia by hanging	13	99	5 h	M	1670
Control #4	-	Head injury	29	305	4 h	M	1011
Control #5	-	Multiple injury	34	71	6 h	M	632
Control #6	-	Meningitis	2	75	11 h	F	103
Control #7	-	Drowning	7	272	12 h	M	4898
Control #8	-	Asthma	1	259	10 h	M	1547
Control #9	-	Drowning	2	286	12 h	F	1791
Control #10	-	Cardiovascular disease	49	160	5 h	M	4915
Control #11	-	Rejection of cardiac transplant	8	214	20 h	F	1706
Control #12	-	Commotio cordis	4	237	17 h	M	4670
Control #13	-	Pneumonia	47	124	5 h	F	4640
AxD #1	K63E	Complication of disease	27	139	22 h	F	5377
AxD #2	N77S	Complication of disease	2	NA	17 h	F	2768
AxD #3	R79C	Complication of disease	13	364	7 h	M	613
AxD #4	R79C	Complication of disease	28	245	18 h	F	5517
AxD #5	E210K	Complication of disease	33	273	20 h	F	M3596
AxD #6	R239C	Complication of disease	2	175	4 h	M	1161
AxD #7	R239C	Complication of disease	6	87	12 h	M	338
AxD #8	R239H	Complication of disease	0	347	4 h	M	1070
AxD #9	R239H	Complication of disease	1	0	7 h	F	5488
AxD #10	S247P	Complication of disease	50	139	17 h	F	4858
AxD #11	E373K	Complication of disease	0	192	18 h	F	885
AxD #12	D395Y	Complication of disease	0	244	2 h	F	1482
AxD #13	D417A	Complication of disease	42	217	4 h	F	5109

PMI, post-mortem interval; NA, not available.

### GFAP cross-linking *in vitro* through cysteine-dependent oxidation

**Figure 2:**
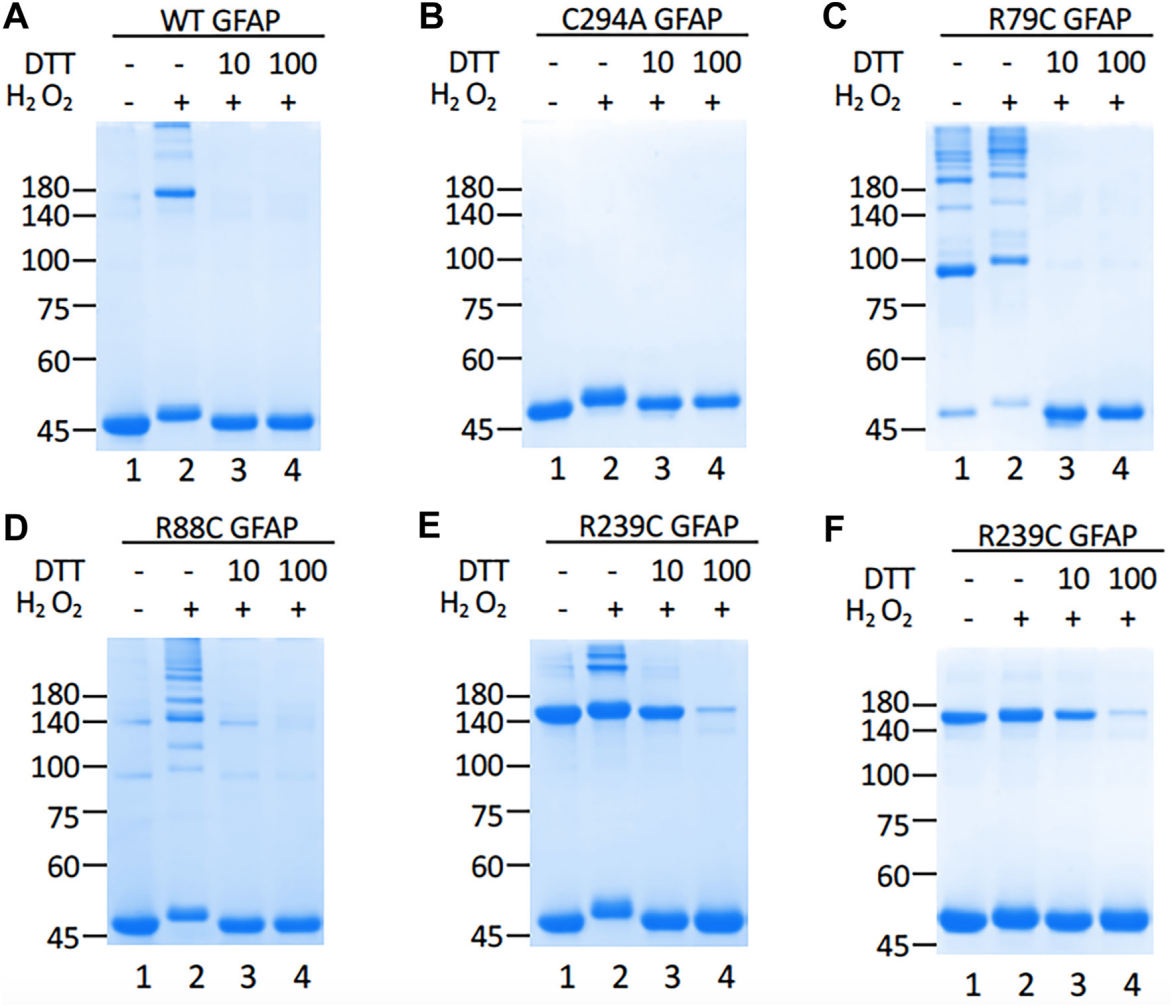
**GFAP oxidation and disulfide bond formation *in vitro*.** Purified recombinant WT (*A*) and mutant (*B*–*F*) GFAP were either untreated (*A*–*F*, lane 1) or treated with 10 mM H_2_O_2_ for 15 min (*A*–*F*, lane 2) as indicated. Peroxide-treated GFAPs were subsequently treated with 10 mM (*A*–*F*, lane 3) or 100 mM (*A*–*F*, lane 4) DTT for 15 min. After treatments, WT and mutant GFAPs were analyzed by SDS-PAGE under either nonreducing (*A*–*E*) or reducing (*F*) conditions, followed by Coomassie blue staining. Molecular weight markers (in kDa) are indicated on the *left*. GFAP, glial fibrillary acidic protein.

Given the dramatic shift of GFAP solubility and molecular weight in the RF fraction of AxD samples, we next determined whether GFAP could be modified by cross-linking *via* cysteine oxidization. To analyze GFAP crosslinking *in vitro*, recombinant human WT GFAP was exposed to H_2_O_2_, a widely recognized second messenger produced in response to oxidative stress through redox signaling (52). Following H_2_O_2_ treatment, a range of HMW GFAP bands was observed under nonreducing conditions (Fig. 2*A*, lane 2). Oxidized GFAP treated with the reducing agent DTT completely collapsed all HMW GFAP into a monomeric form (Fig. 2*A*, lanes 3 and 4), suggesting GFAP crosslinking by disulfide bond formation through cysteine oxidation. To investigate whether cysteine residue is required for peroxide-induced disulfide bond formation, we generated a C294A mutant with the GFAP’s only cysteine residue at position 294 being replaced by alanine. This mutant prevented GFAP from forming HMW crosslinks either in nonreducing conditions (Fig. 2*B*) or in response to peroxide treatment (Fig. 2*B*, lane 2), confirming that the Cys-294 is a critical residue required for oxidative crosslinking of GFAP.

Cystine-generating mutations are quite common in AxD, appearing in the major isoform GFAP-α with a frequency of 16%. To test whether introduction of additional cysteine residues by missense mutations could enhance GFAP cross-linking through extra disulfide bonds, we generated human cystine-generating mutants, R79C, R88C, and R239C GFAPs, which were subjected to a combination of redox treatments as indicated before analyzing by either nonreducing (Fig. 2, *C*–*E*) or reducing (Figs. 2*F*, and S3) conditions. Under nonreducing conditions, R79C GFAP (Fig. 2*C*, lane 1) treated with H_2_O_2_ (Fig. 2*C*, lane 2) formed a smear of HMW bands ranging from ∼100 to 300 kDa with a prominent ∼100 kDa species, which were not detected under reducing conditions (Fig. S3). While R88C GFAP formed ∼100 and 140 kDa bands (Fig. 2*D*, lane 1), peroxide treatment produced a cluster of HMW species (Fig. 2*D*, lane 2) with a banding pattern slightly different from that of R79C mutant (Fig. 2*C*, lane 2). The HMW bands of both R79C and R88C mutants were readily reduced into monomeric forms by DTT treatment (Fig. 2, *C* and *D*, lanes 3 and 4), suggesting they were crosslinked by cysteine-dependent disulfide bond formation.

Among these GFAP mutants, the R239C mutant is unique in that it already formed a band corresponding in size to ∼180 kDa (Fig. 2*E*, lane 1) even when this mutant was analyzed under reducing conditions (Fig. 2*F*, lane 1). Notably, the HMW species were more resistant to reduction by DTT treatment (Fig. 2, *E* and *F*, lane 3), and only when treated with 100 mM DTT did most of the HMW bands collapsed into monomeric 50 kDa band (Fig. 2, *E* and *F*, lane 4). These results suggest that R239C GFAP is in a more oxidized state than R79C and R88C mutants *in vitro*, resulting in the formation of more heavily cross-linked species of GFAP.

### Effect of cystine-generating mutants on GFAP assembly *in vitro*

**Figure 3:**
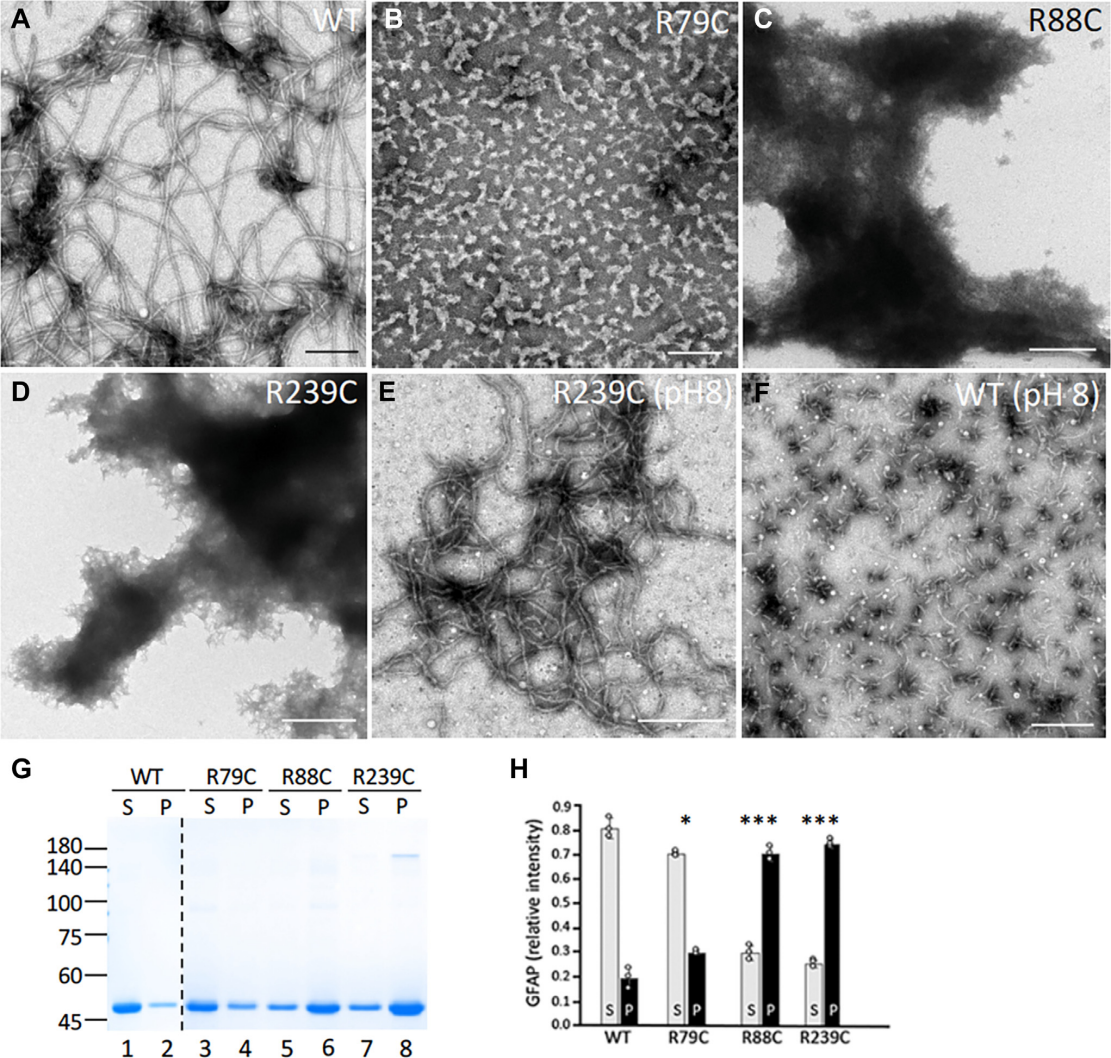
**Assemblyproperties of cysteine-generating GFAP mutants.** Purified WT (*A*, *B*), R79C (*C*), R88C (*F*), and R239C (*D*, *E*) GFAP at a concentration of 0.25 mg/ml were assembled *in vitro*. Before assembly was completed, WT (*A*) and R239C (*D*) GFAP in low ionic strength buffer (pH 8) were fixed and processed for subsequent negative staining. After assembly, GFAPs were processed by negative staining followed by EM (*B*, *C*, *E*, and *F*). Note that R239C mutant showed a greatly increased tendency to polymerize even under preassembly conditions (*D*, pH 8), under which WT GFAP (*A*, pH 8) remained mostly as unit length filament-like structures. Bar represents 500 nm, except in (*E*) and (*F*), which were 1 μm. *G*, WT and mutant GFAPs were subjected to a low-speed sedimentation assay and the resulting supernatant (S) and pellet (P) fractions were analyzed by reducing SDS-PAGE, followed by Coomassie blue staining. Under these assay conditions, WT (lane 1) and R79C (lane 3) GFAP remained mainly in the supernatant fraction. In contrast, R88C (lane 6) and R239C (lane 8) mutants sedimented more efficiently into the pellet fraction. Molecular weight markers (in kDa) are indicated on the *left*. *Dashed* line indicated that samples were run on different gels. *H*, quantification of GFAP mutants in the supernatant and pellet fractions were compared to WT controls. Data are mean ± SD. ∗*p* < 0.05, ∗∗∗*p* < 0.001 (two-tailed *t* test). Each *white* dot represents a biological replicate (n = 3). GFAP, glial fibrillary acidic protein.

Given that cystine-generating mutants in human GFAP readily formed crosslinked species in response to peroxide treatment, we next determine how extra cysteine residue affects the assembly properties of GFAP. WT and mutant GFAP were assembled *in vitro* and visualized by negative staining followed by EM. Whereas WT GFAP assembled into typical 10-nm filaments (Fig. 3*A*), the R79C mutant failed to assemble into 10-nm filaments but formed particle-like structures with irregular diameter (Fig. 3*B*). Both R88C (Fig. 3*C*) and R239C (Fig. 3*D*) GFAP assembled into filamentous-like structures that were prone to aggregation. Notably, the R239C mutant showed a greatly increased tendency to polymerize even under preassembly conditions (Fig. 3*E*), under which WT GFAP remained mostly as short filament pieces that looked like unit-length filaments (Fig. 3*F*). When *in vitro* assembly was performed under nonreducing conditions, both WT and mutant GFAPs failed to assembled into 10-nm filaments. The extent of filament aggregation was assessed by a low-speed sedimentation assay (Fig. 3*G*). Whereas 80 ± 6% WT (Fig. 3*G*, lane 1) and 70 ± 4% R79C (Fig. 3*G*, lane 3) GFAP remain in the supernatant fraction, 70 ± 4% R88C (Fig. 3*G*, lane 6) and 75 ± 3% R239C (Fig. 3*G*, lane 8) mutants were found in the pellet fraction. Quantification results were shown (Fig. 3*H*), indicating a greater tendency of these GFAP mutants to aggregate.

### Cystine-generating mutants formed HMW GFAP crosslinks in astrocytes

**Figure 4:**
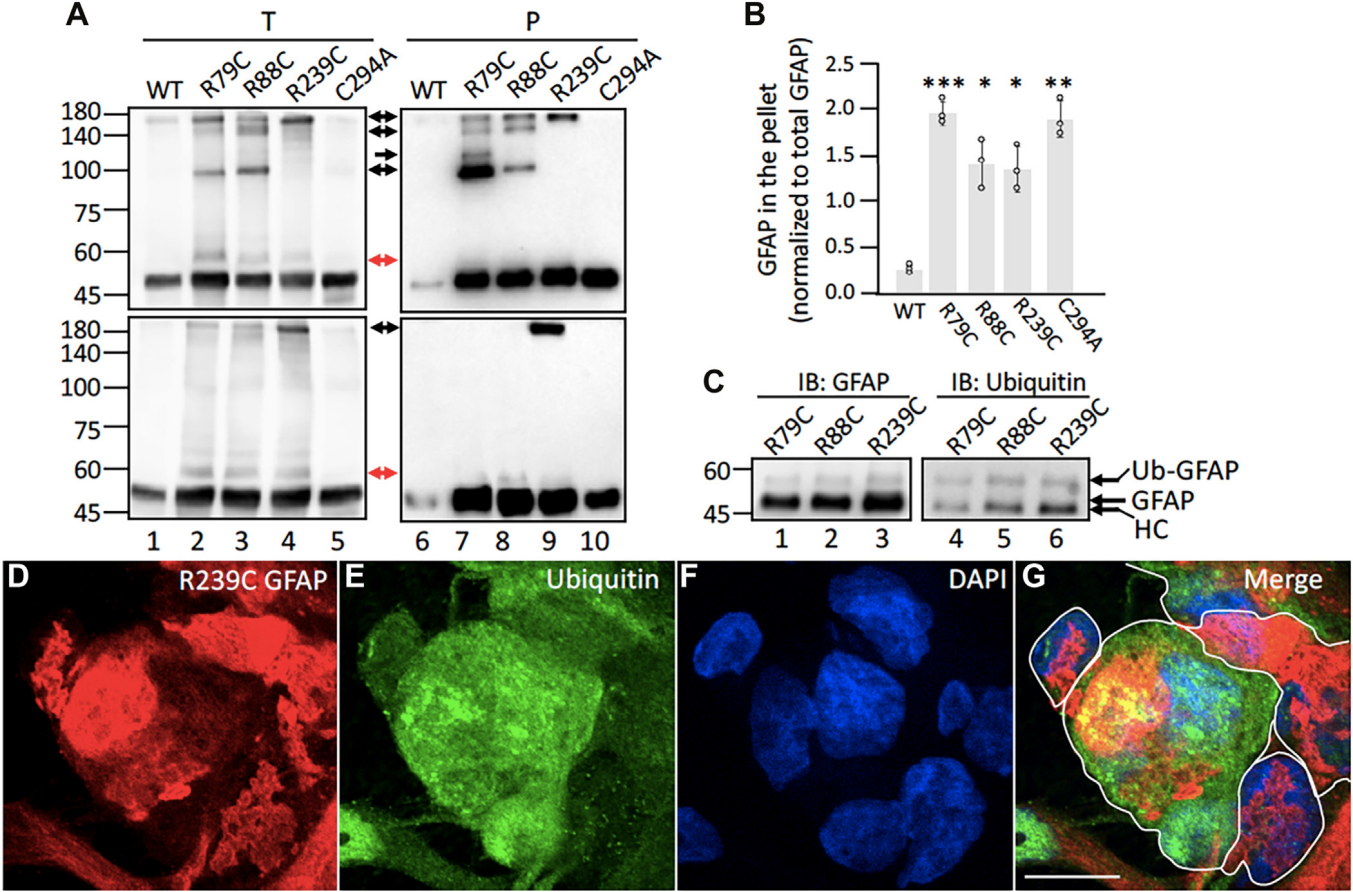
**Cystine-generating GFAP mutants formed cross-linked GFAP species in astrocytes.** Primary astrocytes derived from GFAP KO rats were transduced with indicated GFAP expression constructs. At 72 h after transduction, cells were extracted, and the total (*A*, lanes 1–5) and pellet (*A*, lanes 6–10) fractions were analyzed by either nonreducing (*A*, *top panels*) or reducing (*A*, *bottom panels*) immunoblotting using an anti-GFAP antibody. Note that high molecular weight (HMW) GFAP bands were absent in cells expressing cysteine-deficient C294A GFAP (*A*, lanes 5 and 10). In-gel staining was shown to visualize total protein profiles and to assist comparison of equal protein loading in each sample (Fig. S4*A*). Molecular weight markers (in kDa) are indicated on the *left*. HMW GFAPs are indicated by *black arrows*, and an immunopositive band above the monomeric GFAP is indicated by *red arrows*. *B*, mutant GFAPs in the pellet fraction relative to total GFAP were quantified and compared to WT GFAP. Data are mean ± SD. For all two-tailed *t* test, ∗*p* < 0.05, ∗∗*p* < 0.01, and ∗∗∗*p* < 0.001. Each *white* dot represents a biological replicate (n = 3). *C*, the pellet fractions from cystine-generating mutants-transduced KO astrocytes were subjected to immunoprecipitation using a mouse monoclonal anti-GFAP antibody SMI21, followed by immunoblotting using a rabbit polyclonal anti-panGFAP (lanes 1–3) and a mouse monoclonal anti-ubiquitin (lanes 4–6) antibodies. GFAP and its ubiquitinated form (Ub-GFAP), as well as the heavy chain (HC) of the capture antibody are indicated on the *right*. Molecular weight markers (in kDa) are indicated on the *left*. In-gel staining and full-length blots were shown in Fig. S4*B*. *D*–*G*, GFAP-KO astrocytes transduced with R239C GFAP were double stained with anti-GFAP (*D*, *red* channel) and anti-ubiquitin (*E*, *green* channel) antibodies. Cells were counterstained with DAPI (*F*, *blue* channel) to reveal nuclei. A merged image was shown (*G*), with *white* lines indicating the edge of GFAP-transduced cells. Note that ubiquitin labeling was prevalent in astrocytes but did not always colocalize with GFAP, which may reflect accumulation of other ubiquitinated proteins. Bar represents 10 μm. Representative images were shown from astrocyte cultures prepared from three GFAP-KO rats. GFAP, glial fibrillary acidic protein.

We next determined if disulfide cross-linking of GFAP affects its solubility properties in astrocytes. To accomplish this, primary astrocytes derived from GFAP KO rats were used. These cells, which do not express any endogenous GFAP that could complicate the interpretation, were transduced with either human WT or mutant GFAPs. At 72 h after transduction, cells were extracted using an extraction protocol that solubilized nonaggregated forms of GFAP but retained GFAP aggregates (53). The total lysates (Fig. 4*A*, lanes 1–5) and pellet fraction (Fig. 4*A*, lanes 6–10) were analyzed by either nonreducing (Fig. 4*A*, top panels) or reducing (Fig. 4*A*, bottom panels) immunoblotting using an anti-GFAP antibody. Whereas WT GFAP was extracted almost completely from WT GFAP-transduced cells (Fig. 4*A*, lane 6), all the GFAP mutants were more resistant to extraction (Fig. 4*A*, lanes 7–10) and remained mainly in the pellet fraction (Fig. 4*B*). Under nonreducing condition, HMW GFAP bands with sizes ranging from ∼100 to 200 kDa were detected mainly in the pellet fraction in astrocytes transduced with cystine-generating mutants (Fig. 4*A*, lanes 7–9, top panel), but not in those transduced with WT (Fig. 4*A*, lane 6) and C294A (Fig. 4*A*, lane 10) GFAP. Similar results were observed in human AxD patients carrying cystine-generating GFAP mutants (Fig. S4*C*). Notably, R239C mutant formed a ∼200 kDa cross-linked species even under reducing conditions (Fig. 4*A*, lane 9, bottom panel).

We noticed that in cells expressing the cystine-generating mutants, an immunopositive band above the prominent GFAP band was detected exclusively in the pellet fraction (Fig. 4*A*, lanes 7–9, red arrows). Based on the reduced solubility and increased molecular weight, the upper band might represent GFAP that is potentially modified by ubiquitination. To test this possibility, insoluble fractions prepared from astrocytes transduced with the cystine-generating GFAP mutants were subjected to immunoprecipitation using a mouse monoclonal anti-GFAP antibody, followed by immunoblotting using anti-GFAP (Fig. 4*C*, lanes 1–3) and anti-ubiquitin (Fig. 4*C*,lanes 4–6) antibodies. The upper band was immunopositive for both GFAP and ubiquitin (Fig. 4*C*, Ub-GFAP), confirming the presence of ubiquitinated GFAP in the pellet fraction. Immunofluorescence studies further showed that GFAP aggregates (Fig. 4*D*) were also immunopositive for ubiquitin (Fig. 4*E*) when astrocytes were transduced with R239C GFAP (Fig. 4*G*).

### GFAP ubiquitination in the human patients and rodent AxD models

**Figure 5:**
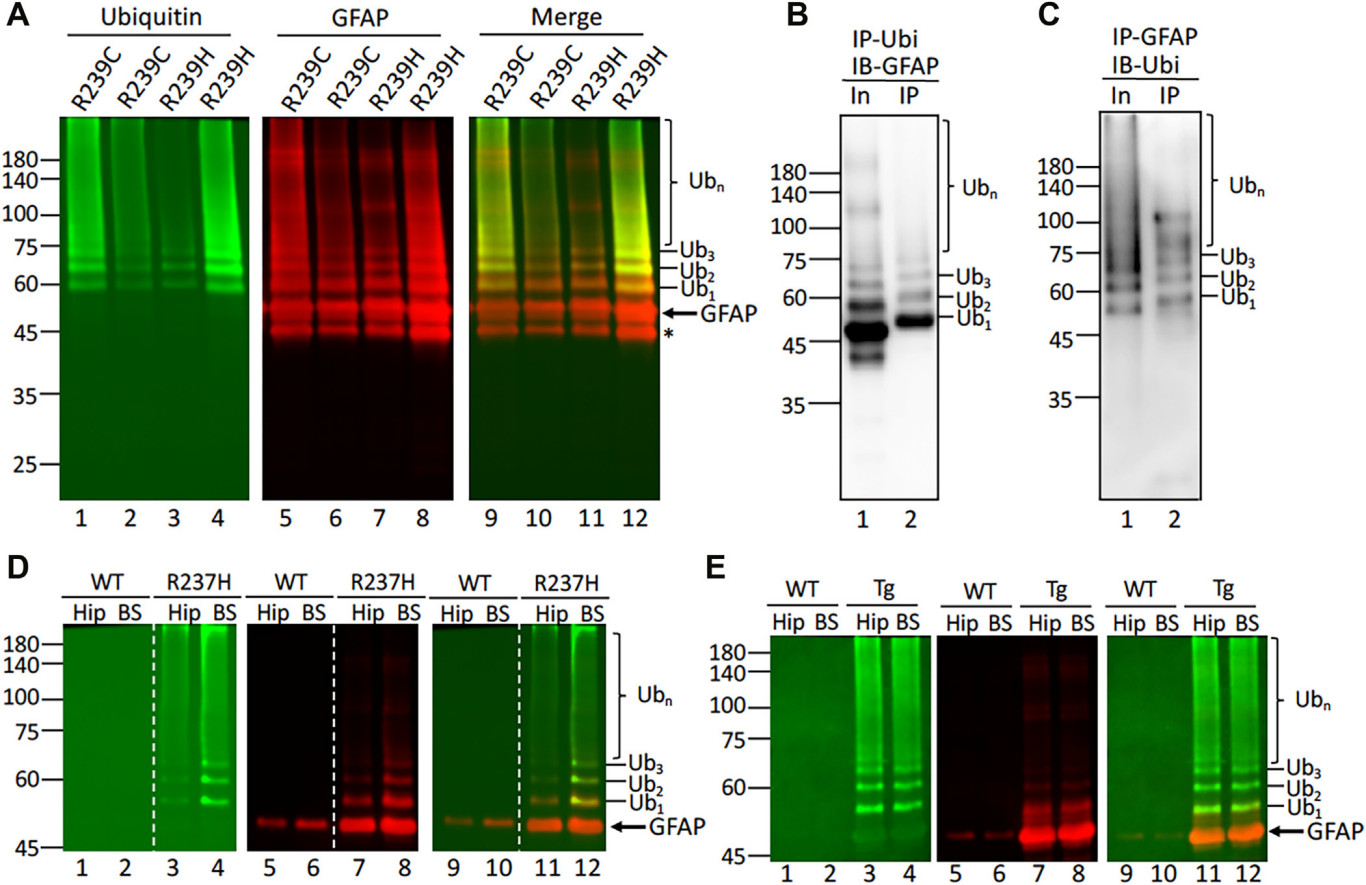
**GFAP is pathologically ubiquitinated in human AxD brains and rat AxD model.***A*, RF-enriched fractions from four AxD cases carrying either R239C or R239H mutations were analyzed by immunoblotting using anti-ubiquitin (lanes 1–4) and anti-panGFAP (lanes 5–8) antibodies. Immunoblots with merged signals showing high molecular weight smears of GFAP species (Ub_1-3_, and Ub_n_) were immunopositive for both ubiquitin and GFAP. Total protein profiles of the RF-enriched fraction were visualized by in-gel staining with trichloroethanol (Fig. S5*A*). *B* and *C*, RF-enriched fraction from an AxD patient with R239C mutation was subjected to immunoprecipitation using either anti–ubiquitin (*B*) or anti–GFAP (*C*) antibody. The inputs (In, lane 1) and immunoprecipitates (IP, lane 2) were analyzed by immunoblotting using anti-panGFAP (*B*) and anti-ubiquitin (*C*) antibodies. Ubiquitinated GFAPs were indicated on the *right*, with Ub_1-3_, are presumably mono-, di-, and tri-ubiquitinated GFAP and Ub_n_ represents polyubiquitinated GFAP. Molecular weight markers (in kDa) are shown on the *left*. Similar accumulation of ubiquitinated GFAP species were also observed in different brain regions of AxD rodent models. *D*, RF-enriched fractions prepared from hippocampus (Hip) and brain stem (BS) of WT and R237H rat brains were analyzed by immunoblotting using anti-ubiquitin (lanes 1–4) and anti-GFAP (lanes 5–8) antibodies. Merged images showed ubiquitinated GFAP (lanes 9–12) in the indicated brain regions of R237H rat. Representative immunoblots were shown from samples prepared from three WT and R237H rats at 8 weeks of age. *Dashed* line indicated that lanes were run on the same gel but were noncontiguous. *E*, immunoblotting analysis of RF-enriched fractions from WT and GFAP^Tg^ mice for ubiquitin (1, 2, 3, 4) and GFAP (lanes 5–8) in the hippocampus (Hip) and brain stem (BS). A merged image showed ubiquitinated GFAP in the indicated brain regions (lanes 9–12). Each lane represents samples prepared from individual animals at 8 weeks of age (n = 3). Molecular weight markers (in kDa) are indicated on the *left*. In-gel staining was shown (Fig. S5, *C* and *D*) to assist comparison of equal protein loading. GFAP, glial fibrillary acidic protein; RF, Rosenthal fiber.

We next determined whether GFAP is ubiquitinated in patients with AxD. Analysis of RF fractions from four type I AxD cases by immunoblotting revealed a series of higher MW bands that were clearly visualized by both the anti-ubiquitin (Fig. 5*A*, lanes 1–4) and anti-GFAP (Fig. 5*A*, lanes 5–8) antibodies with similar patterns (Fig. 5*A*, lanes 9–12). To demonstrate directly that GFAP was ubiquitinated, an AxD sample from RF fraction was subjected to reciprocal immunoprecipitation using anti-ubiquitin (Fig. 5*B*) and anti-GFAP (Fig. 5*C*) antibodies. Subsequent immunoblotting revealed a series of HMW species that were immunopositive for both GFAP and ubiquitin (Fig. 5, *B* and *C*, lane 2). Interestingly, we found that the minor isoform GFAP-δ was not modified by ubiquitination (Fig. S5*B*).

To investigate whether GFAP ubiquitination is a common pathological event in the setting of disease, we analyzed the RF-enriched fraction prepared from brains of the AxD model rats with an R237H mutation in the endogenous *gfap* (Fig. 5*D*). The AxD rats exhibited hallmark pathology with GFAP aggregation in the form of RFs (54). To determine whether insoluble GFAP was ubiquitinated and whether different brain regions showed differences in this modification, RF-enriched fractions were prepared from hippocampus (Hip) and brain stem of WT and R237H rats. Immunoblotting revealed that ubiquitinated GFAP was detected in both brain regions of AxD rats (Fig. 5*D*, lanes 11 and 12), but not in WT controls (Fig. 5*D*, lanes 9 and 10). Similar results were observed in GFAP transgenic (Tg) mice (15), where ubiquitinated GFAP species were also detected in the RF-enriched fractions prepared from hippocampus and brain stem (Fig. 5*E*, lanes 11 and 12).

### Arginine increased the solubility of aggregation-prone mutant GFAP

**Figure 6:**
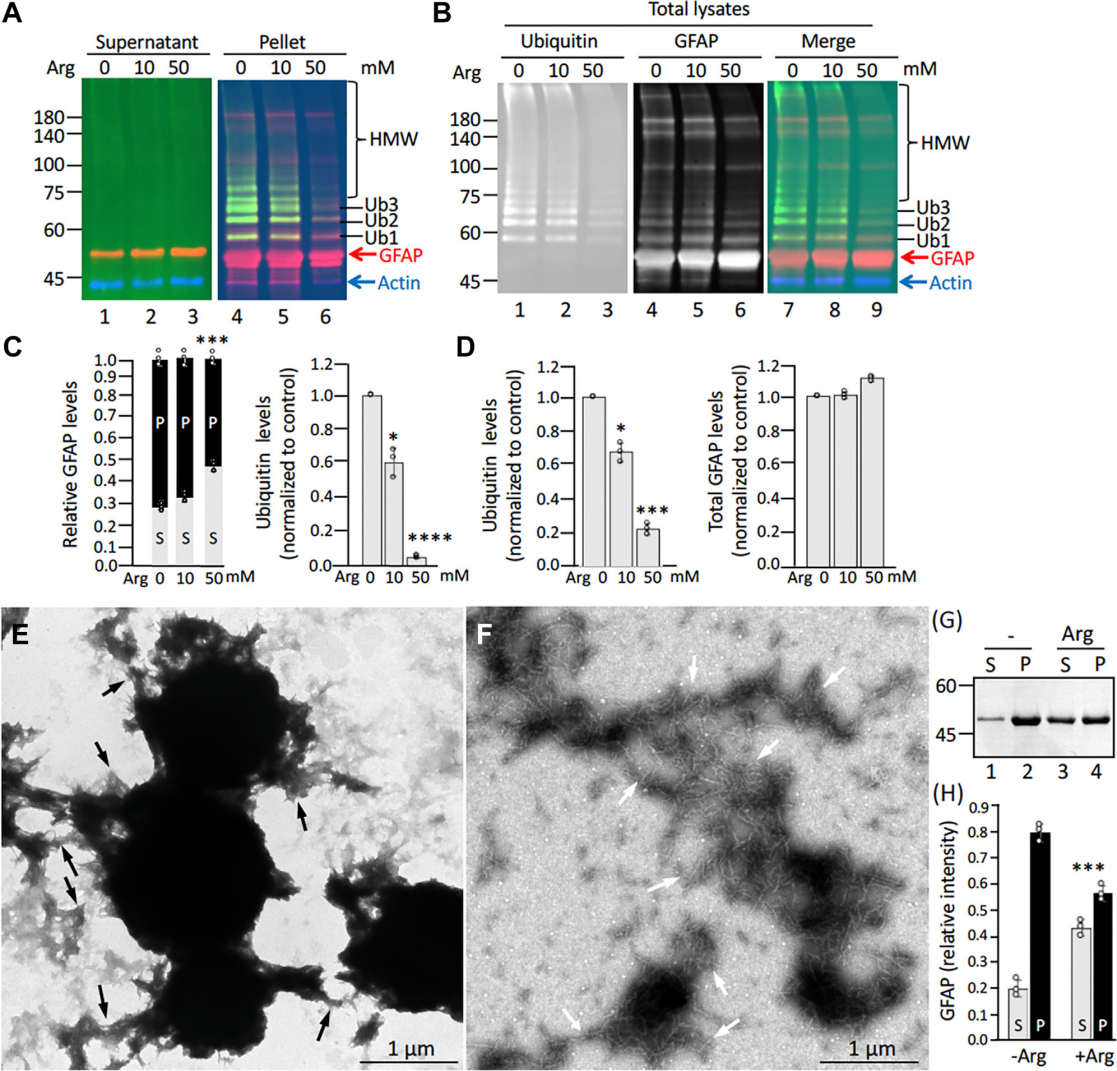
**Effect of arginine on GFAP solubility and ubiquitination.***A*, SW13 (Vim-) cells were transduced with E373K GFAP in the presence of 10 mM or 50 mM arginine as indicated. At 48 h after transduction, cells were extracted the resulting supernatant (*A*, lanes 1–3) and pellet (*A*, lanes 4–6) fractions, as well as the total lysates (*B*) were analyzed by immunoblotting using anti-ubiquitin (*green* channel) and anti-GFAP (*red* channel) antibodies. Merged immunoblot was shown (*A*, and *B* lanes 7–9). Molecular mass markers are shown on the *left*, and the positions of GFAP and ubiquitinated GFAP species are indicated on the *right*. In-gel staining was shown (Fig. S6*C*) to assist comparison of equal protein loading of each lane. Quantification of GFAP and ubiquitin levels in the pellet (*C*) and total (*D*) fractions of arginine-treated cells compared to untreated controls. Data are mean ± SD. For all two-tailed *t* test, ∗*p* < 0.05, ∗∗∗*p* < 0.001, and ∗∗∗∗*p* < 0.0001. Each *white* dot represents a biological replicate (n = 3). *E*–*H*, purified recombinant E373K GFAP at a concentration of 0.25 mg/ml was assembled *in vitro* in the absence (*E*) or presence (*F*) of 50 mM arginine. Assembled GFAPs were negatively stained and visualized by transmission electron microscopy. Bar represents 1 μm. The extent of GFAP aggregation was assessed by a low-speed sedimentation assay (G). The E373K GFAP assembled *in vitro* in the absence (lanes 1 and 2) or presence (lanes 3 and 4) of 50 mM arginine was subjected to a low speed centrifugation, and the resulting supernatant (lanes 1 and 3) and pellet (lanes 2 and 4) fractions were analyzed by SDS-PAGE and visualized by Coomassie blue staining. Molecular weight markers (in kDa) are indicated on the *left*. *H*, quantification of arginine-treated mutant GFAP in the supernatant and pellet fractions compared to untreated controls. Data are mean ± SD. ∗∗∗*p* < 0.001 (two-tailed *t* test). Each *white* dot represents a biological replicate (n = 3). GFAP, glial fibrillary acidic protein; HMW, high molecular weight.

Given that abnormal ubiquitination of GFAP is accompanied by its aggregation and a shift in biochemical solubility, we next determined whether increase in GFAP solubility could decrease GFAP aggregation and ubiquitination. For this study, we used arginine, a well-established chemical chaperone with an aggregation-modulating property (55), to evaluate its potential effects on GFAP solubility and ubiquitination. We selected the E373K GFAP for our study because this mutation involves a substitution of a highly conserved glutamate by lysine, a possible ubiquitination site in GFAP. Indeed, ubiquitinated GFAP was readily detected in the RFs of an AxD case carrying the E373K mutation (Fig. S6*A*). Similarly, ubiquitinated GFAP species were observed in the pellet fraction of primary astrocytes transduced with the E373K mutant (Fig. S6*B*). To test the effect of arginine on the solubility property of E373K GFAP, SW13(−) cells that do not express any cytoplasmic IFs were used, since treatment of primary astrocytes with arginine caused detachment of these cells. SW13(−) cells transduced with E373K GFAP in the absence or presence of arginine were extracted with buffers that solubilized nonaggregated forms of GFAP while retaining the GFAP aggregates. Immunoblotting analysis of the supernatant and pellet fractions revealed that ∼72% E373K GFAP remained in the pellet fraction (Fig. 6*A*, lane 4), with only ∼28% mutant protein in the supernatant fraction (Fig. 6*A*, lane 1). In the presence of 50 mM arginine, mutant-expressing cells exhibited a decrease in GFAP level to ∼53% in the pellet fraction (Fig. 6*A*, lane 6) while increased GFAP level to ∼47% in the supernatant fraction (Fig. 6*A*, lane 3). Since ubiquitination occurred in the pellet fraction (Fig. 6*C*), we analyzed ubiquitin level in this fraction. Analysis of E373K GFAP-transduced cells revealed that arginine treatment resulted in a dramatic decrease in ubiquitin to a level ∼5% (Fig. 6*A*, lane 6) of untreated cells (Fig. 6*A*, lane 4). In the total cell lysates, a similar decrease in ubiquitin level (Fig. 6*D*) was observed in arginine-treated cells (Fig. 6*B*, lane 3) compared to untreated controls (Fig. 6*B*, lane 1). The level of GFAP (Fig. 6*D*) was comparable between arginine-treated (Fig. 6*B*, lane 6) and untreated (Fig. 6*B*, lane 4) mutant-expressing cells, indicating that arginine could decrease ubiquitination of GFAP by increasing its solubility rather than by decreasing its levels.

To further investigate the extent of GFAP aggregation in the presence of arginine, we performed *in vitro* assembly studies. EM showed that after assembly, E373K GFAP formed large, irregular shaped aggregates (Fig. 6*E*) from which abnormal filaments with irregular width occasionally protruded (Fig. 6*E*, arrows). In the presence of 50 mM arginine, however, less aggregated materials comprising short filamentous structures (Fig. 6*F*, arrows) were observed. The extent of GFAP aggregation was further assessed by a low-speed sedimentation assay. On its own, most of the E373K GFAP sedimented into the pellet fraction (Fig. 6*G*, lane 2), with only 19 ± 4.6% (Fig. 6*H*) remaining soluble (Fig. 6*G*, lane 1). In the presence of 50 mM arginine, however, the mutant protein in the supernatant fraction (Fig. 6*G*, lane 3) was dramatically increased (44 ± 3.7%, Fig. 6*H*), suggestive of a potential anti-aggregation effect of arginine.

## Discussion

### Cystine-generating GFAP mutants are susceptible to oxidative crosslinkings

We begin our study by the unexpected findings that when cystine-generating mutants, R79C, R88C, and R239C GFAP were purified recombinantly from bacteria, they consistently formed HMW oligomers even when these proteins were analyzed under reducing conditions by standard SDS-PAGE. These observations suggest that the cystine-generating mutants have a strong tendency to be oxidized nonenzymatically *in vitro* and form crosslinked species through disulfide bond formation. Subsequently, we showed that cystine-generating mutants were highly susceptible to oxidative modification. Treatment of these GFAP mutants with oxidative stressors such as H_2_O_2_ resulted in a formation of a wide range of disulfide cross-linked GFAP species, suggesting cysteine-dependent GFAP crosslinking as a dominant response to oxidative stress. In human AxD patients and rodent AxD models, GFAP in RFs readily formed HMW GFAP crosslinks with patterns similar to those observed *in vitro*. These findings suggest that cysteine-dependent oxidation could contribute to GFAP aggregation in pathological situations associated with oxidative stress. Previous studies using autopsy samples from AxD patients showed that astrocytes containing RFs are immunoreactive for advanced lipid peroxidation and glycation end-products (41, 42), providing further evidence in support of the role of oxidative stress in the pathological aggregation of GFAP. While cystine-generating GFAP mutants were susceptible to cysteine-dependent crosslinking, WT amino acid residues replaced by AxD mutations in GFAP, such as histidine and tyrosine, might produce new targets for other types of crosslinking through side chain oxidations (56), which may contribute to the diversity and complexity of GFAP proteoforms that could alter the functional properties of GFAP in response to fluctuating oxidative conditions in astrocytes.

Among the cystine-generating mutants we have analyzed in this study, the R79C GFAP is unique in that this mutation prevented GFAP assembly *in vitro*. The reason why the R79C mutation is so disruptive remains unknown, but a previous study has shown that mutations within the highly conserved LNDR sequence spanning amino acids 76 to 79 of human GFAP impeded filament assembly drastically (57). Under our *in vitro* assembly conditions, the R79C GFAP only formed roundish particles (Fig. 3*B*). Similar observations were made on corresponding mutations in other IF proteins, such as R89C keratin 18 (58), R113C vimentin (59), and R117C desmin (60), demonstrating that this arginine residue is of general importance for IF assembly. Change at the R79 in the LNDR motif of GFAP can be severe, probably because it resides at the *g* position of the first heptad (*abcdefg*) that is part of the coil 1A initiator region of IF protein. Based on the structural study of vimentin, a type III IF protein closely related to GFAP, substitution of the R79 with cysteine, may potentially disrupt the intrahelical salt bridge between R79 and E75 of GFAP, thereby compromising the stability of the tetrameric complexes in a way that prevents proper longitudinal annealing of the unit length filaments into extended intermediate filaments (60).

The R239C GFAP has previously been studied in detail, but the results produced were not very consistent. In a cell-based study, Hsiao *et al.* showed that both WT and R239C GFAP formed filamentous IF networks when expressed in Cos-7 cells or primary astrocytes (61). In other studies using the same cell types (20, 62, 63), however, the same mutant formed aggregates at a significantly higher frequency than WT GFAP. Although Hisao *et al.* reported that the R239C mutant formed GFAP filaments similar to those formed by WT protein *in vitro*, a clear distinction was observed in its resistance to high salt extraction (61). In our study, the R239C mutant showed a greatly increased tendency to polymerize *in vitro* even under low ionic strength buffer conditions, in which WT GFAP remained mainly as unit-length filaments. These results suggest the extra cysteine residue in GFAP may promote its assembly and subsequent aggregation through enhanced interfilament interactions. This finding may provide an explanation for the otherwise puzzling observations that although R239C GFAP was assembly-competent both *in vitro* and in transfected cells, this mutation was found to increase the resistance of GFAP to extraction under elevated salt concentrations (61). Thus, although the R239C mutation did not appear to affect filament formation *per se*, this mutation induced an over-assembly of GFAP filaments that could alter their solubility properties and filament organization through an increased interfilament interaction.

### Redox-dependent regulation of GFAP oxidation

GFAP aggregation as a result of mutation-induced crosslinking through cysteine oxidation may contribute to increased oxidative stress and altered redox signaling. Indeed, previous studies of brain tissues from GFAP overexpressing and R236H mutant GFAP mice showed a marked oxidative stress response and the induction of several oxidative stress response genes (64, 65) through the binding of Nrf2 to a common antioxidant response element. A further potential consequence of oxidative stress that is particularly relevant for astrocytes in AxD is a compromise in mitochondrial function. Studies of transfected cells showed that mutant GFAPs do exert this effect. In particular, GFAP R239C expression induced a more oxidized cellular status, with increased mitochondrial superoxide generation (44). We found that R239C mutant already formed HMW GFAP crosslinks when transduced into primary astrocytes, providing additional evidence to support that the expression of mutant GFAP induced a more oxidized environment, which could contribute to cellular oxidative distress and cysteine-dependent GFAP oxidation. Thus, our data suggest that oxidation of cysteines in GFAP through disulfide bond formation may represent a rapid and reversible regulatory switch to modulate the functional properties of GFAP in response to oxidative stress.

Numerous neurodegenerative diseases are characterized by the formation of intracellular aggregates (66), in which alterations in disease-associated PTMs also occurs. For instance, TAR DNA-binding protein of 43 kDa (TDP-43), a component of RFs, was pathologically modified by ubiquitination and phosphorylation in the brains of AxD patients (23). Misfolded mutant TDP43 found in familial ALS and FTLD patients with TDP-43 mutations forms aberrant cysteine cross-links, which accumulate as insoluble TDP43 aggregates (67). Strikingly, we observed a similar phenomenon for cystine-generating GFAP mutants that accumulated in the insoluble aggregates comprising distinct disulfide cross-linked species in the AxD brains. Although the exact role of disulfide species in either GFAP or TDP43 aggregation is currently unclear, both TDP-43 and GFAP are redox-sensitive target proteins. The similarities in redox sensitivity between GFAP and TDP-43 proteins suggest a common underlying mechanism in which redox-regulated oxidative modification promotes cross-linking of similarly aggregation prone proteins.

### GFAP is pathologically modified by ubiquitination in AxD

In addition to cross-linked GFAP oligomers, we observed GFAP-immunopositive bands sized between 75-50 kDa that are common to AxD samples. The identity of these upper bands remains unknown, and our previous data suggest that they were not GFAP isoforms (53). Given that aberrantly modified GFAP is usually accompanied by a shift in solubility and molecular weight, the HMW GFAP species detected in the RF fractions in human patients and AxD models suggest GFAP could be modified by PTMs potentially involving ubiquitination. Previous studies have shown that αB-crystallin is ubiquitinated in RFs (45, 68), but whether GFAP itself carries this modification has not been tested experimentally. Our biochemical analysis showed a distinct AxD-specific GFAP signature characterized by the accumulation of ubiquitinated GFAP species, suggesting that ubiquitination of GFAP is a common event in the setting of disease. However, we do not know whether WT, mutant GFAP, or both were ubiquitinated. Nor can we be certain whether GFAP becomes ubiquitinated as an intermediate in the turnover of the protein, or whether ubiquitinated GFAPs in RFs are only seen as part of a disease process. The specific sites of ubiquitylation in GFAP are also not known, aside from putative lysine residues identified through previous mass spectrometric analyses (69).

It is well known that ubiquitination of IF proteins is often difficult to detect for numerous technical reasons (70), including specific deubiquitinase activity (71). Perhaps our success in detecting discrete bands of ubiquitinated GFAP species in brain tissues where others have failed (23, 24, 45, 51, 57) might be related to two reasons. First, we used a specific monoclonal anti-ubiquitin antibody FK2 that recognized both mono- and poly-ubiquitin conjugates, but not free ubiquitin. Our results showed that this antibody detected mono-, di-, and tri-ubiquitinated GFAPs with sizes ranged between 75-50 kDa, which were migrated away from the higher MW smear of ubiquitinated proteins typically seen in the ubiquitin immunoblots. Second, we prepared RF-enriched fractions from AxD brain tissues using a sequential extraction protocol to increase the detection specificity and sensitivity for minor pathological forms of GFAP. This particular approach allowed us to detect the endogenous ubiquitinated GFAPs without relying on transfection of tagged ubiquitin to increase ubiquitin conjugates (62). However, it is possible that the ubiquitinated GFAPs normally exist but at very low levels and that their detection in the context of disease might simply reflect the detection threshold that was exceeded when insoluble GFAPs were highly enriched in the RF fraction. We found the ubiquitinated GFAP species were accumulated mainly in the RF fraction, suggesting ubiquitination could play an important role in modulating GFAP solubility. In support of this role, we found that arginine, an aggregation-modulating chemical chaperone (72), could increase the biochemical solubility of an aggregation-prone AxD mutant by decreasing GFAP ubiquitination and aggregation. Although the mechanisms in which arginine can act as a suppressor of GFAP aggregation remained unexplored, our data suggest that reduction of cellular stress response and maintaining astrocyte function by decreasing GFAP aggregation could be beneficial for individuals with AxD.

The presence of ubiquitinated GFAPs in the RF fractions of human AxD brains and rodent AxD models suggests impaired proteasomal degradation in astrocytes. However, if ubiquitin conjugation plays a major role in the degradation of GFAP, then it seems paradoxical that ubiquitinated GFAP species accumulate. One possible explanation is that the GFAP crosslinking by cysteine-dependent oxidation could become a poor substrate for proteasomal degradation. Thus, crosslinked GFAP oligomers may be particularly effective at inhibiting proteasome activity. In support of this role, previous studies have shown that decreasing larger GFAP oligomers to smaller oligomers or monomers with αB-crystallin restored proteolytic activity of proteasome (73). These observations add to the recent view that the oligomeric species may be the biologically more toxic forms than larger aggregates in other neurodegenerative proteinopathies (74). Another possible explanation is that defects in proteasome degradation may occur when GFAP levels were highly elevated in the context of AxD (63). Support of this possibility comes from our findings that the presence of ubiquitinated GFAP species in AxD samples correlated with the highest insoluble GFAP burden. These observations suggest that whereas ubiquitination of GFAP is a normal physiological process that is involved in its turnover, proteasome inhibition as a result of increased GFAP oligomerization is accompanied by GFAP ubiquitination. The third possibility is that the GFAP mutants may exert their inhibitory effects by direct binding to the proteasome complex, which could block the entrance of ubiquitinated substrates into the inner catalytic compartment. However, whether GFAP mutants bind to specific proteasomal components or whether these bindings alter the active sites of the catalytic subunits for proteolysis will require further study.

### A pathogenic cycle initiated by GFAP mutations

**Figure 7:**
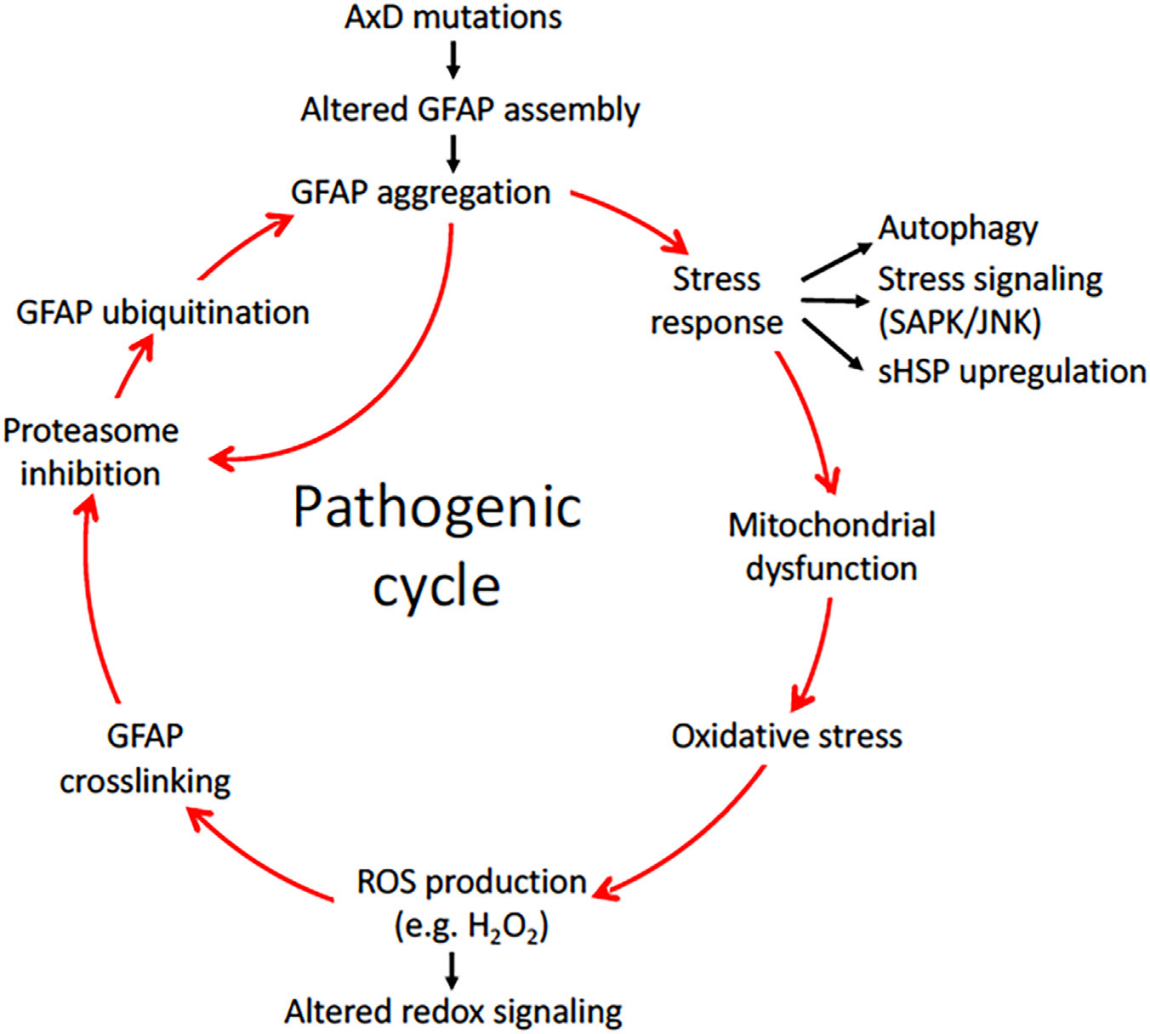
**We hypothesize a potential two-step mechanism, in which GFAP aggregation induced by AxD mutations through altered filament assembly may represent an initiation event that triggers subsequent GFAP oxidation and ubiquitination, leading to proteasome inhibition and further aggregation.** This eventually would lead to a pathogenic cycle that emerge with further disease progression in the pathogenesis of AxD. GFAP, glial fibrillary acidic protein.

The results from our findings, together with evidence from previous studies, suggest a series of pathogenic events leading to AxD, involving interplay between GFAP aggregation and aberrant modifications by GFAP ubiquitination and oxidation (Fig. 7). GFAP aggregation initiated by AxD mutations through altered assembly process could induce a cellular stress response leading to mitochondrial dysfunction and oxidative stress (44). This could elicit GFAP oxidation through increased production of reactive oxygen species (ROS). Once this occurs, HMW GFAP species formed by oxidative crosslinking may lead to proteasome inhibition and GFAP ubiquitination. This in turn constitutes a positive feedforward loop leading to further proteasome inhibition and GFAP aggregation. In yet another positive feedforward circuit, GFAP aggregation reduce proteasome activity, which in turn increases GFAP ubiquitination and further aggregation. An irreversible point in disease progression may be reached whereby pathological changes initiated by AxD mutations could lead to a more generalized disruption of GFAP proteostasis. This model is consistent with the ideal that expression of mutant GFAP is the initiating event in AxD (75), and any subsequent downstream changes would then act to exacerbate the disease process. Figure 7 incorporates these ideas into a diagram of the events that we believe to be important in the pathogenesis of AxD.

In conclusion, our study suggests a potential two-step mechanism, which involves a bidirectional crosstalk between GFAP aggregation and modifications leading to a pathogenic cycle in AxD. The increasing number of post-translational modifications regulating GFAP assembly and filament properties suggests that complex regulatory mechanisms exist to maintain and fine-tune the functional properties of GFAP filaments. Future studies will shed light on any cross-talk between these regulatory mechanisms and potential synergism in promoting GFAP modifications in the degenerative brain diseases.

## Experimental procedures

### Human brain tissues and preparation of RF-enriched fraction

De-identified post-mortem fresh-frozen and fixed AxD patients and non-AxD control brain tissues were provided by the NIH NeuroBiobank and are described in a previous publication (37) and the Table 1 of the current study. Use of post-mortem human tissue for this study was approved by the University of North Carolina at Chapel Hill Institutional Review Board and abided by the Declaration of Helsinki principles. All AxD cases had typical neuropathological features, including multiple RFs. Post-mortem intervals were comparable between AxD cases and non-AxD controls and were <24 h in all cases. To prepare GFAP-enriched RF fraction, brain tissues were extracted at 0.1 g/ml (w/v) sequentially with Triton buffer (20 mM Tris–HCl, 5 mM EDTA, 140 mM NaCl, 1% (v/v) Triton X-100), high-salt buffer (Triton buffer supplemented with 1.5 M KCl), sucrose buffer (0.85 M sucrose, 20 mM Tris–HCl, and 5 mM EDTA), and urea buffer (6 M urea, 20 mM Tris–HCl, and 5 mM EDTA). All buffers contained cocktails of protease inhibitors (10 μM ALLN, 2 μg/ml leupeptin, 5 μg/ml aprotinin, and 2 mM PMSF, all from Sigma-Aldrich) and phosphatase inhibitors (5 mM sodium fluoride, 1 mM sodium vanadate, 1 mM sodium pyrophosphate, and 1 mM β-glycerol phosphate, all from Sigma-Aldrich). The urea insoluble pellets were resuspended in the TES buffer (20 mM Tris-HCl, pH 7.4, 5 mM EDTA, 1% (w/v) SDS) and sonicated at 4 °C for 30 s. The whole extraction procedure was performed with the addition of 10 mM β-mercaptoethanol to all buffers to prevent nonspecific oxidation of proteins during the extraction process. Protein concentrations of each fraction were determined by bicinchoninic acid (BCA) assay (Thermo Fisher Scientific Inc.) using bovine serum albumin (BSA) as a standard.

### EM of human brain tissues

Post-mortem brain tissues from AxD patients were cut and fixed directly in fixative solution, containing 3% (w/v) glutaraldehyde and 2% (w/v) paraformaldehyde (both from Electron Microscopy Sciences) in sodium cacodylate buffer (0.1 M pH 7.4) for 1 h at room temperature and then stored in fixative solution at 4 °C overnight. After being washed thrice with cacodylate buffer, tissues were post-fixed with 1% (w/v) osmium tetroxide at 4 °C for 1 h, followed by staining with 4% (w/v) uranyl acetate (Electron Microscopy Sciences) for 2 h. After being washed several times with distilled water, fixed tissues were subjected to a series of graded ethanol dehydration, followed by overnight incubation with 1:1 propylene oxide:epoxy resin (Agar Scientific). After two changes with 100% fresh resin, tissues were transferred to BEEM capsules (Agar Scientific) and were polymerized in fresh resin at 60 °C overnight. Ultrathin sections were cut with a Leica UCT Ultramicrotome and collected on formvar and carbon-coated nickel grids (Ted Pella Inc.). The specimens on grid were stained with 4% (w/v) uranyl acetate for 30 min followed by staining with Reynold’s lead citrate for additional 30 min. The sections were examined under a HT-7700 electron microscope (Hitachi Technologies) operating at 100 kV.

### Rodent models of AxD

All animal experiments were approved by the Institutional Animal Care and Use Committee of the College of Life Sciences at the National Tsing Hua University (NTHU IACUC Approval No. 109088 and 111060) and in accordance with the guideline of Agriculture Guidebook for the Care and Use of Laboratory Animals. *Gfap* knockout and R237H *gfap* knock-in rats were generated by Prof. Albee Messing’s group (Waisman Center, University of Wisconsin-Madison) using CRISPR-Cas9-based mutagenesis to introduce a *gfap*-null mutation and a R237H mutation that is homologous to the common R239H mutation of human AxD in the endogenous rat *gfap* gene (54). Rats carrying the desired mutations were confirmed by PCR analysis of DNA isolated from tail biopsies, using a pair of primers that flank exon 4 (Table 2). Both male and female heterozygous *gfap*+/R237H rats at 8-week-old were used for experiments, and sex-matched and age-matched WT littermates were used as controls. Transgenic mice carrying several copies of normal human *gfap* transgene were maintained in the FVB/N background as hemizygotes (15). Genotyping was performed by isolating the genomic DNAs from tail biopsies of postnatal day 1 (P1) mice, followed by PCR analysis using a pair of primers (Table 2). RF-enriched fractions were prepared from specific regions of rat and mouse brain using the same fractionation protocol as described for human brain tissues before being analyzed further by immunoblotting.

**Table 2:** Primers used for genotyping

Gene Name	Primer	Primer sequence (5′ to 3′)	Purposes
Human GFAP	Forward	AAGACCGTGGAGATGCGGGATGGA	Amplify human GFAP from Tg mice
	Reverse	GGGAGCTCAGGTCTGGGGAAATG	
Rat GFAP	Forward	GAGAGAGATTCACACACAA	Amplify GFAP fragment from mutant allele
	Reverse	CACTGAGCAAACTGGTGAGC	
Rat GFAP	Forward	AGAGAGATTCGCACTCAG	Amplify GFAP fragment from WT allele
	Reverse	GCATCAAAAAGCAGGCTCTC	
Primers for mutagenesis			
GFAP R79C	Forward	CAATGACTGCTTTGCCAGCTAC	Introducing an R to C substitution in GFAP
	Reverse	GCAAAGCAGTCATTGAGCTCCAT	
GFAP R88C	Forward	GAAGGTTTGCTTCCTGGAACA	Introducing an R to C substitution in GFAP
	Reverse	AGGAAGCAAACCTTCTCGATG	
GFAP R239C	Forward	AGAGATCTGCACGCAGTATGA	Introducing an R to C substitution in GFAP
	Reverse	TGCGTGCAGATCTCTTTCAG	
GFAP R239H	Forward	AGAGATCCACACGCAGTATGA	Introducing an R to H substitution in GFAP
	Reverse	TGCGTGTGGATCTCTTTCAG	
GFAP C294A	Forward	CTTGACCGCCGACCTGGAGTC	Introducing an C to A substitution in GFAP
	Reverse	AGGTCGGCGGTCAAGGACTGC	

### Plasmid construction and site-directed mutagenesis

Sets of *gfap* mutations with nucleotide changes encoding specific amino acids were generated by InFusion HD Plus Cloning System (Takara Bio) using the full-length human *gfap* in the pET23b vector (62) or pLEX vector (76) as a template. The nucleotide sequence of all newly constructed vectors was confirmed by Sanger’s DNA sequencing.

### Expression and purification of recombinant GFAPs

For bacteria expression of GFAP, pET23b expression vectors encoding either WT or mutant GFAPs were transformed into Novagen’s *Escherichia coli* BL21 pLysS strain (Merck Millipore). Recombinant protein expression was induced by the addition of 0.5 mM IPTG when the optical density OD_595_ reached at least 0.2. After 4 h postinduction, bacteria were collected by centrifugation at 6000*g* for 30 min at 4 °C. Overexpressed GFAP formed inclusion bodies, which were processed as described (77). The final pellet consisting predominantly of GFAP were extracted in the urea buffer (6 M urea, 20 mM Tris–HCl, pH 8, 5 mM EDTA, 1 mM PMSF) at 4 °C overnight. After centrifugation at 80,000*g* for 20 min at 4 °C, the urea-soluble fractions were incubated with 0.05% (v/v) PEI to precipitate bacterial DNA. After centrifugation, the GFAP-enriched supernatant was collected and further purified by ion exchange chromatography using an AKTAprime plus system (GE Healthcare) equipped with a DEAE Sephacel column. GFAP was eluted from the column with a linear gradient of 0 to 0.5 M NaCl in the urea buffer over 1 h at a flow rate of 1 ml/min. Where indicated, GFAP were further purified by a CM Sepharose column (GE Healthcare). Column fractions were analyzed by SDS-PAGE, followed by Coomassie blue staining, and those containing purified GFAP were aliquoted and stored at −80 °C. Protein concentrations were determined by BCA assay using BSA as a standard.

### *In vitro* assembly, sedimentation assay, and EM

Purified GFAP diluted to 0.25 mg/ml in 6 M urea in a buffer of 10 mM Tris–HCl, pH 8, 5 mM EDTA, and 10 mM β-mercaptoethanol were dialyzed stepwise against 3 M urea in the same buffer for 4 h, and then against the same buffer without urea at 4 °C overnight. Filament assembly was completed by dialyzing against assembly buffer (10 mM Tris–HCl, pH 7.0 and 50 mM NaCl, 10 mM β-mercaptoethanol, final pH 6.95–7.05) at 30 to 32 °C for 12 to 16 h. In some experiments, *in vitro* assembly was performed under nonreducing conditions using the same buffers without β-mercaptoethanol. The filament-forming efficiency was assessed by high-speed sedimentation assay (78), where the assembly mixture was layered onto a cushion of 0.85 M sucrose in the assembly buffer and centrifuged for 20 min at 80,000*g* at 20 °C using a CS150NX tabletop micro-ultracentrifuge (Hitachi Koki Co.). To assess the extent of GFAP aggregation in the assembly mixture, samples were subjected to a low-speed centrifugation at 3000*g* for 5 min at room temperature. The resulting supernatant and pellet fractions were mixed with SDS sample buffer in volumes proportional to the original sample volumes, and they were analyzed by SDS-PAGE, followed by Coomassie Blue staining. The distribution of GFAP between pellet and supernatant fractions were analyzed using a ChemiDoc MP Imaging System (Bio-Rad) and quantified by the ImageLab Software (v. 6.1, Bio-Rad).

After assembly, GFAPs were spread on the carbon-coated copper grids (Ted Pella Inc.) for 1 min and excess sample was removed by blotting with a filter paper. The grids were rinsed with distilled water and then stained with 1% (w/v) uranyl acetate (Electron Microscopy Sciences) for 5 min. The grids were then examined under a HT-7700 transmission electron microscope (Hitachi High-Tech) operating at 100 kV. Images were acquired using a CCD camera before being processed further for figures using the Adobe Photoshop CC (Adobe Systems).

### Immunoblotting analyses

Immunoblotting was performed using the wet electrophoretic transfer system (Bio-Rad) as previously described (53). After electrophoretic transfer of proteins, nitrocellulose membranes (Pall Life Sciences) were blocked with 3% (w/v) BSA in Tris-buffered saline with Tween (TBST; 20 mM Tris-HCl, pH 7.4 and 150 mM NaCl, containing 0.1% (v/v) Tween 20) at room temperature for at least 1 h. After several washes with TBST, membranes were incubated with primary antibody (Table 3) at 4 °C overnight, followed by incubation with horseradish peroxidase-conjugated anti-mouse, anti-rabbit or anti-rat secondary antibodies (Table 3) for at least 1 h. The blots were developed with Enhanced Chemiluminescence substrate (Western Lightning, PerkinElmer Life Sciences), and signal produced were digitized using a ChemiDoc MP Imaging System (Bio-Rad Laboratories, Inc). For multiplex fluorescent immunoblotting, goat anti-mouse IgG and anti-rabbit IgG conjugated with either StarBright Blue 520 or StarBright Blue 700 were used as secondary antibodies (Bio-Rad). Signals from nonsaturated exposures of immunoblots were quantified using the ImageLab Software (Bio-Rad). Where indicated, in-gel staining by trichloro ethanol (Sigma-Aldrich), which reacted specifically with the tryptophan at position 256 of GFAP to produce fluorescent signal, was used to reveal the total protein profiles in the analyzed samples.

**Table 3:** Summary of antibodies used for immunoblotting and immunostaining

Antibody (clone no.)	RRID	Host	Assay dilution	Supplier/Reference
GFAP (SMI21)	AB_509978	Mouse	IB: 1:5000	BioLegend
GFAP-α	AB_10672298	Mouse	IB: 1:5000	NeuroMab
GFAP-δ		Rabbit	IB: 1:5000	In house (53)
GFAP	AB_2631098	Rabbit	IB: 1:5000	Cell signaling Technology
GFAP	AB_10013482	Rabbit	IB: 1:10,000 IF: 1:1000	Dako
GFAP (GA5)	AB_721051	Mouse	IB: 1:5000	Sigma-Aldrich
Vimentin (V9)	AB_609914	Mouse	IB: 1:5000	Sigma-Aldrich
Vimentin	AB_10695459	Rabbit	IB: 1:5000	Cell Signaling Technology
αB-crystallin	AB_1659585	Mouse	IB: 1:5000	Enzo Life Sciences
Actin	AB_787885	Mouse	IB: 1:5000	Novus
Actin (Rhodamine-conjugated)	AB_2861334	Mouse	IB: 1:10,000	Bio-Rad
Ubiquitin (P4D1)	AB_331292	Mouse	IB: 1:5000	Cell Signaling Technology
Ubiquitin (FK2)	AB_2043482	Mouse	IB: 1:5000 IF: 1:500	Sigma-Aldrich
Secondary antibodies				
2nd antigen (conjugate)	RRID	Host	Assay dilution	
Mouse IgG (HRP)	AB_10015289	Goat	IB: 1:5000	Jackson ImmunoResearch Laboratory
Rabbit IgG (HRP)	AB_2313567	Goat	IB: 1:5000	
Rat IgG (HRP)	AB_2338128	Goat	IB: 1:5000	
Mouse IgG (StarBright 700)	AB_2884948	Goat	IB: 1:10,000	Bio-Rad
Rabbit IgG (StarBright 520)	AB_2884949	Goat	IB: 1:10,000	
Mouse IgG (Alexa Fluor 488)	AB_141607	Donkey	IF: 1:500	Thermo-Invitrogen
Rabbit IgG (Alexa Fluor 594)	AB_141637	Donkey	IF: 1:500	

IB, immunoblotting; IF, immunofluorescence.

### Immunoprecipitation

For immunoprecipitation, the insoluble proteins in the pellet fraction was resuspended in TES buffer (20 mM Tris-HCl, pH 7.4, 5 mM EDTA, 1% (w/v) SDS) by sonication, and then diluted 1:10 in the RIPA buffer without SDS (1% Triton X-100, 0.5% sodium dexoycholate, 5 mM EDTA, 150 mM NaCl, 50 mM Tris, pH 8.0). The resulting solutions were precleared by incubating with Protein G Sepharose (GE Healthcare) for 1 h at 4 °C. After preclearing, samples were incubated with either anti-human GFAP antibody SMI-21 or anti-ubiquitin antibody FK2. The immunocomplex was captured by Protein G Sepharose at 4 °C overnight. After a low speed centrifugation, bound proteins were eluted from the precipitates with SDS sample buffer without β-mercaptoethanol (10 mM Tris, pH 6.8, 1 mM EDTA, 1% SDS, 10% glycerol) at 70 °C to minimize the dissociation of IgG heavy and light chains from the immunocomplex. Eluted proteins were then analyzed further by either non-reducing or reducing SDS-PAGE and immunoblotting as described above.

### Cell cultures

Astrocyte-enriched primary cultures were prepared from either homozygotes *gfap*-null rats or wild-type controls using standard procedures as described previously (76). Briefly, cerebral cortices from postnatal day 1 pulps were dissected in Hank's balanced salt solution (HBSS) followed by incubation with 0.25% (w/v) trypsin at 37 °C for 15 min. After incubating with DNase I (Sigma-Aldrich) for additional 5 min, the cortices were mechanically dispersed by triturating with a Pasture pipette. Dissociated cells were collected by centrifugation at 1000*g* for 5 min, followed by resuspension in plating medium (minimal essential medium (MEM)) containing 5% (v/v) horse serum, 5% (v/v) fetal bovine serum, 100 U/ml penicillin, and 100 μg/ml streptomycin. After filtration through a 70 μm nylon mesh (Greiner Bio-One), cells were plated onto poly-D-lysine-coated plates or dishes at 5 × 10^4^ cells/cm^2^. Cells were cultured for 6 to 7 days with medium change every 2 days. Human adrenal carcinoma SW13 (Vim-) cells were cultured in Dulbecco's modified Eagle's medium (DMEM) supplement with 10% (v/v) fetal bovine serum, 2 mM L-glutamine, 100 U/ml penicillin, and 100 μg/ml streptomycin. Unless otherwise stated, all reagents used for cell culture were purchased from Thermo Fisher Scientific. All cells were cultured at 37 °C in a humidified incubator of 95% (v/v) air and 5% (v/v) CO_2_.

### Lentiviral transduction

Lentiviruses were produced by transiently co-transfecting *gfap*-containing pLEX vector with the psPAX2 packaging (#12260; Addgene) and pMD2.G envelope (#12259; Addgene) vectors into 293T cells (Thermo Fisher Scientific) as described previously (76). Cell transduction was performed by incubating lentiviruses with cultured cells in the presence of 8 μg/ml polybrene (Sigma-Aldrich). At 4 h postinfection, the virus-containing medium was replaced with fresh culture medium. Under these conditions, approximately 80% of cells were infected as determined by immunofluorescence microscopy.

### Immunofluorescence microscopy

Cells fixed in 4% paraformaldehyde (Electron microscopy Science) in PBS were permeabilized with 0.2% (w/v) Triton X-100 (Sigma-Aldrich) in PBS for 5 min, and blocked with 10% (v/v) normal goat serum (Jackson ImmunoResearch Laboratories) in PBS for 1 h at room temperature. After several washes with PBS, cells were incubated with primary antibodies (Table 3) at room temperature for at least 1 h, followed by incubation with secondary antibodies conjugated with Alexa Fluor 488 or Alexa Fluor 594 (Thermo Fisher Scientific) for 1 h. Nuclei were visualized by staining with 4′,6-diamino-2-phenylindole (DAPI). After immunostaining, cells were visualized by a Zeiss LSM800 laser scanning confocal microscope (Carl Zeiss) using either 20 × (0.7 NA) Plan-Neofluar or 40 × (1.3 NA) Apochromat objective lens. Images were acquired by the Zen software (Ver. 2.3) taking 0.5 μm optical sections and processed for figures using Adobe Photoshop CC (Adobe Systems). For quantification of GFAP aggregation, several random fields from at least three coverslips were analyzed by visual assessment of the percentage of transduced cells that displayed GFAP-positive aggregates.

### Subcellular fractionation

Cells plated in 6-well plates (5 × 10^5^ cells) were Dounce homogenized in 300 μl of ice-cold RIPA buffer (50 mM Tris pH 8.0, 150 mM NaCl, 1% NP-40, 5 mM EDTA, 0.5% sodium deoxycholate, and 0.1% SDS) containing 1 mM phenylmethylsulfonyl fluoride and a mixture of protease inhibitors and phosphatase inhibitors as described above. A small aliquot of homogenates was taken as total cell lysates, and the remaining homogenates were centrifuged at 16,000*g* for 15 min at 4 °C. The supernatants were saved as RIPA-soluble fraction for further analysis. The remaining pellets, taken as RIPA-insoluble fraction, were resuspended and sonicated in TES buffer (20 mM Tris–HCl, pH 7.4, 5 mM EDTA, 1% (w/v) SDS) with or without β-mercaptoethanol. Protein concentration of all fractions was determined by the BCA assay (Thermo Scientific Inc.) prior to analysis by immunoblotting.

### Statistical analysis

All quantitative measurements were presented as mean ± SD. Two-tailed unpaired *t* tests were used for comparison between control and experiment groups. For all statistical analyses, the data were considered statistically different if *p* < 0.05.

## Data availability

All data associated with this study are present in the paper and in the supporting information. All other data provided in this article are available from the corresponding author on reasonable request.

## Supporting information

This article contains supporting information.

## Conflicts of interest

The authors declare that they have no conflicts of interests with the contents of this article.
