# Supplementary material for: Glial fibrillary acidic protein is pathologically modified in Alexander disease
Source: J Biol Chem. 2024 May 21;300(7):107402. doi: 10.1016/j.jbc.2024.107402 (PMC11259701; doi:10.1016/j.jbc.2024.107402)
Supplement: Supporting Figures [file mmc1.docx]

**
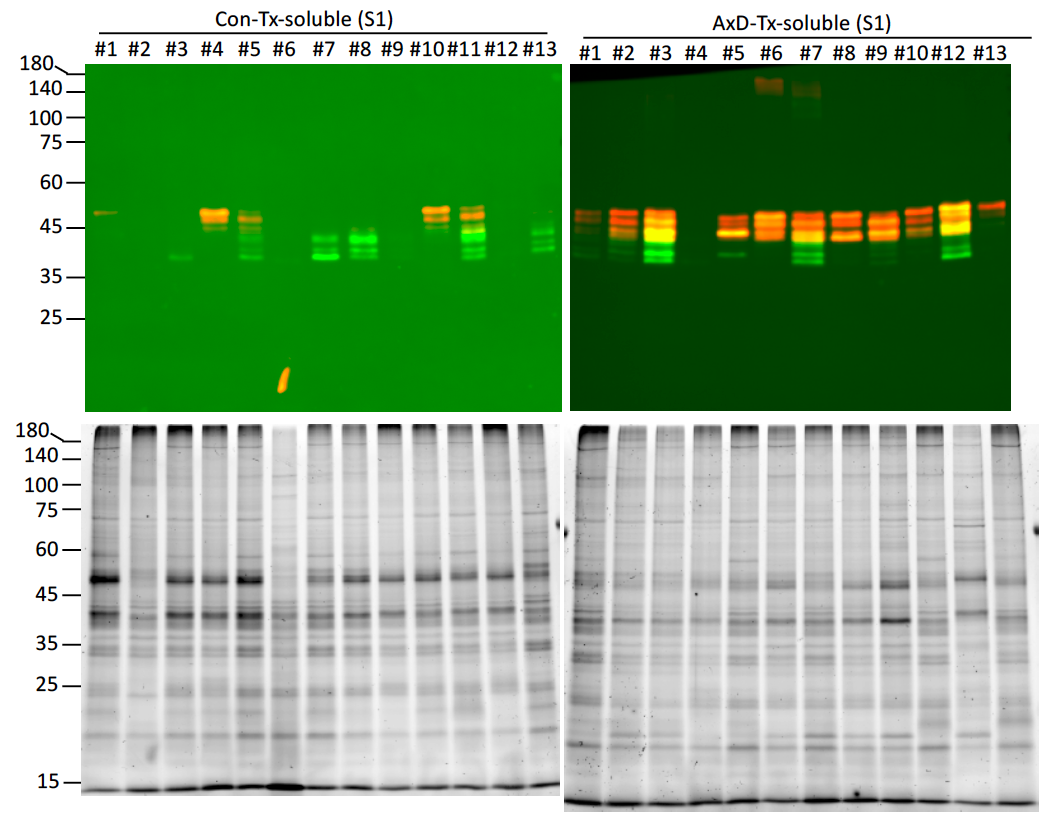
**

**SFig. 1.** The Triton X-100 soluble fractions from non-AxD controls (A, #1-13) and AxD brain tissues (B, #1-13) were analyzed by immunoblotting using a monoclonal anti-GFAP antibody SMI-21 (green channel) and a polyclonal anti-panGFAP antibody (red channel). Merged immunoblot showed the superimposition of both the green and red signals. Total protein profiles of each lane was shown by in-gel staining (A and B, bottom panel). Molecular weight markers (in kDa) are indicated on the left.

**
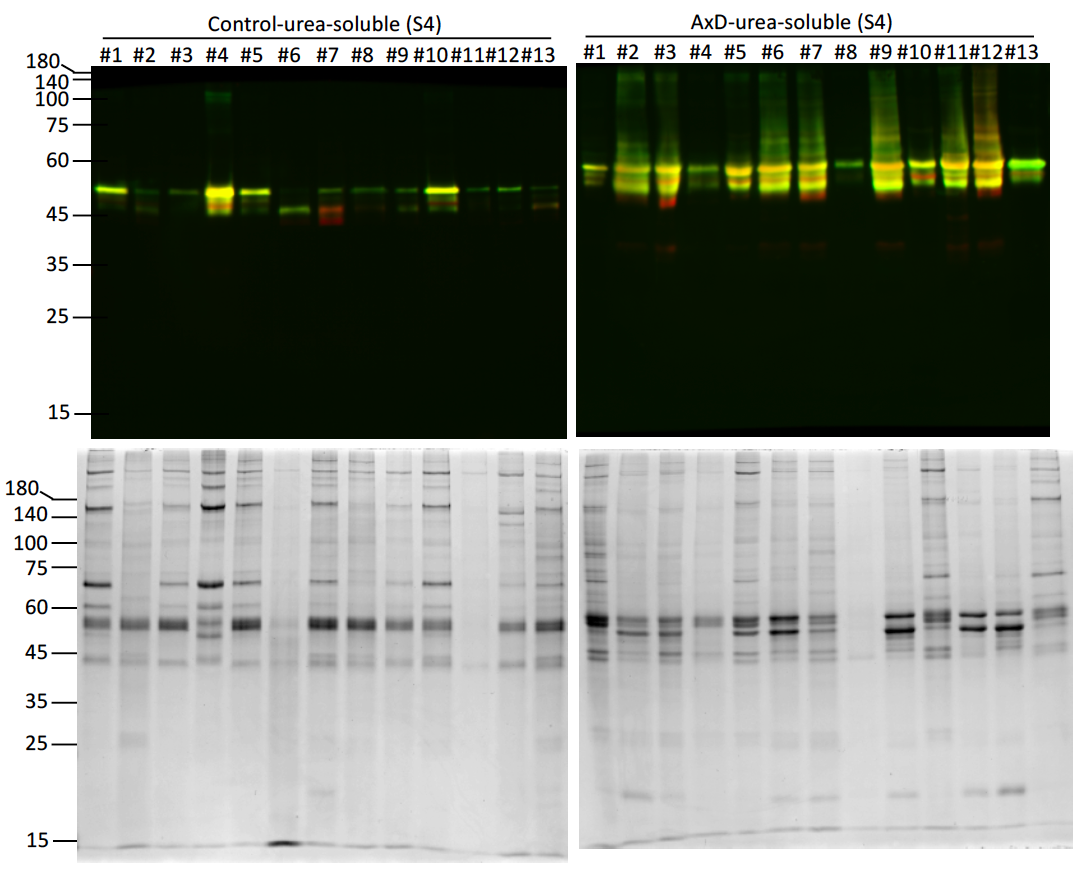
**

**SFig. 2.** The urea soluble fractions from non-AxD controls (A, #1-13) and AxD brain tissues (B, #1-13) were analyzed by immunoblotting using a monoclonal anti-GFAP antibody SMI-21 (green channel) and a polyclonal anti-panGFAP antibody (red channel). Merged immunoblot showed the superimposition of both the green and red signals Total protein profiles of each lane was shown by in-gel staining (A and B, bottom panel). Molecular weight markers (in kDa) are indicated on the left.


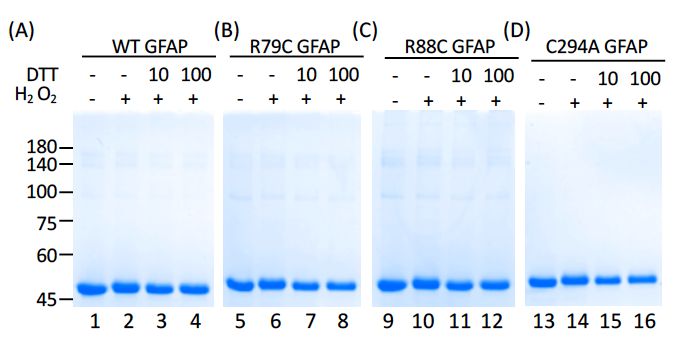


**SFig. 3.** GFAP oxidation and disulfide bond formation in vitro. Purified recombinant WT (A) and mutant (B-D) GFAP were either untreated (A-D, lane 1) or treated with 10 mM H_2_O_2_ for 15 min (A-D, lane 2) as indicated. Peroxide-treated GFAPs were subsequently treated with 10 mM (A-D, lane 3) or 100 mM (A-D, lane 4) DTT for 15 min. After treatments, WT and mutant GFAPs were analyzed by SDS-PAGE under reducing conditions, followed by Coomassie blue staining. Molecular weight markers (in kDa) are indicated on the left.


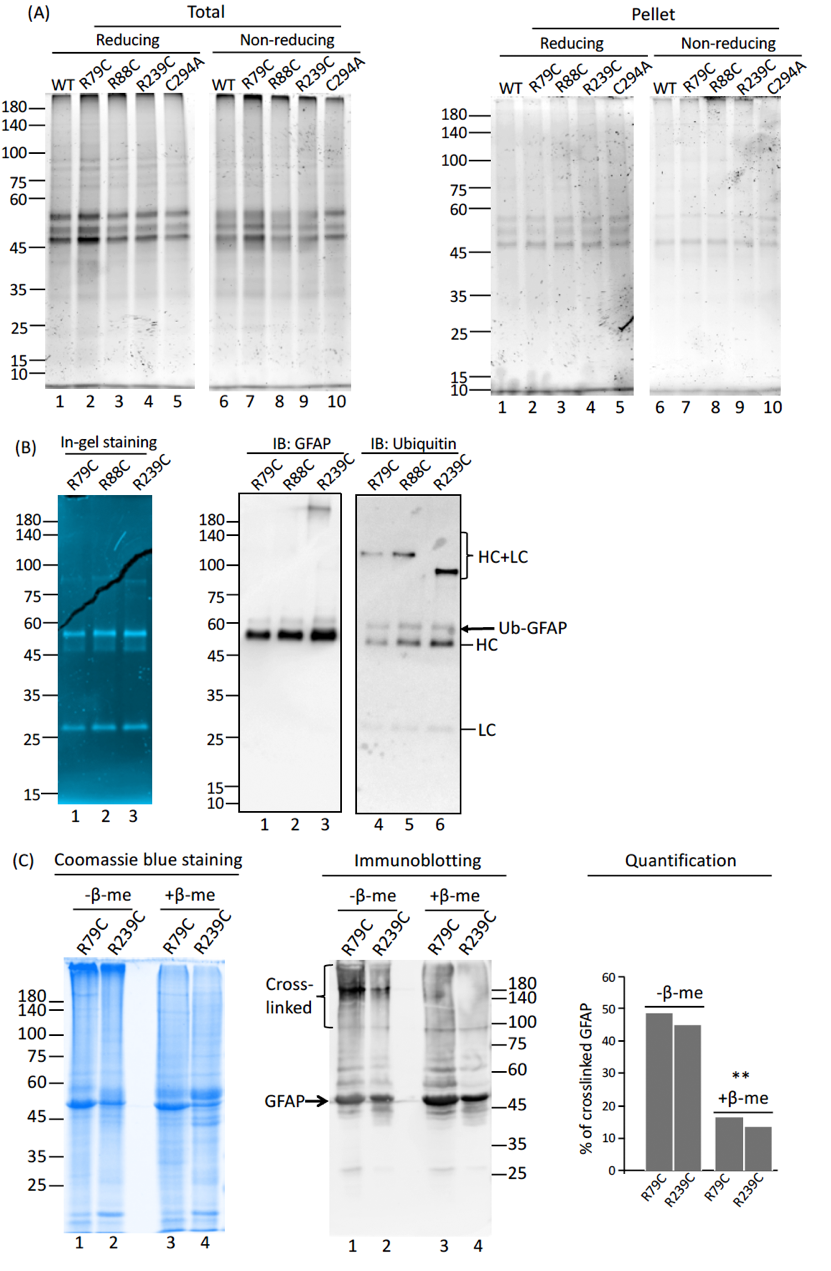


**SFig. 4.** Primary astrocytes derived from GFAP knockout (KO) rats were transduced with indicated GFAP expression constructs. At 72 h after transduction, cells were extracted and the total (A, lanes 1-5) and pellet (A, lanes 6-10) fractions were analyzed by either non-reducing (A, left panel) or reducing (A, right panel) SDS-PAGE followed by in-gel staining. (B) The pellet fractions from GFAP KO astrocytes transduced with cysteine-generating mutants were subjected to immunoprecipitation using a mouse monoclonal anti-GFAP antibody SMI21. Protein samples were analyzed by SDS-PAGE, followed by in-gel staining and immunoblotting using anti-ubiquitin and anti-GFAP antibodies. (C) Rosenthal fiber-enriched fractions prepared from AxD patients carrying either R79C or R239C mutation were analyzed by non-reducing (-β-me) or reducing (+β-me) SDS-PAGE followed by Coomassie blue staining and immunoblotting. GFAP levels in the non-reducing conditions were quantified and compared to GFAP levels in the reducing conditions. **p<0.01 (two-tailed t-test, n=2).

**
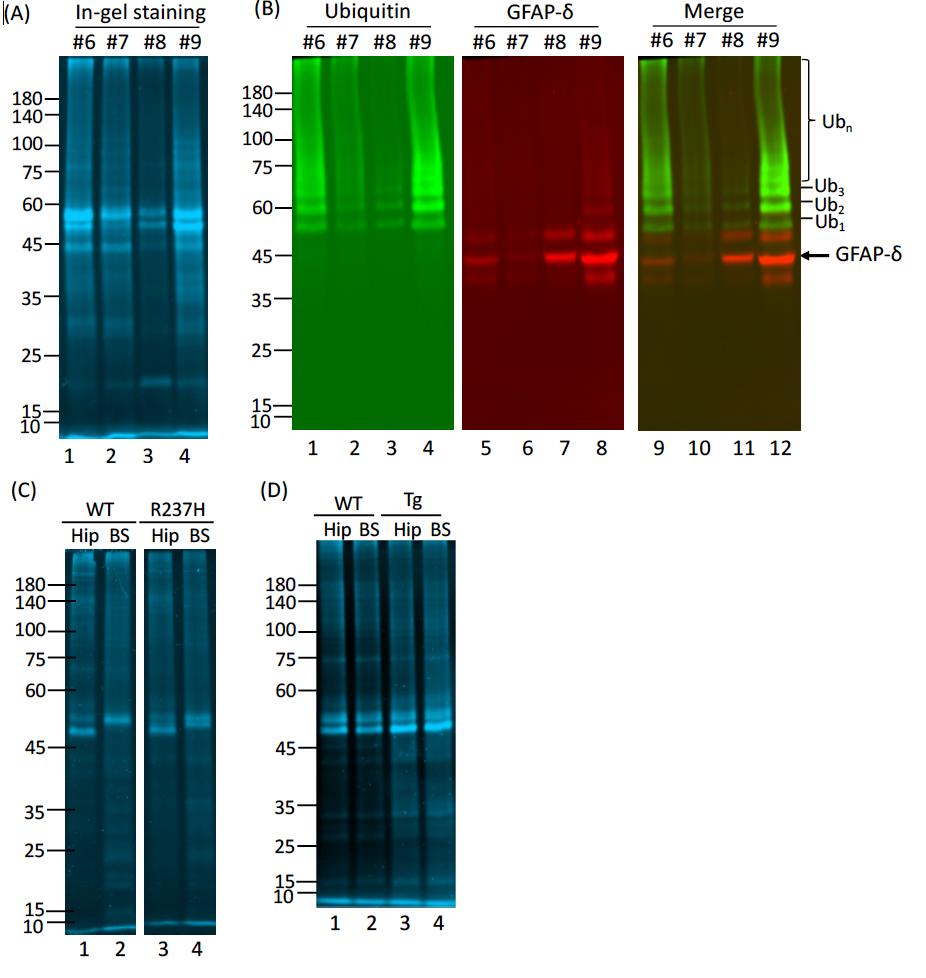
**

**SFig. 5.** GFAP-δ was not ubiquitinated in AxD brains. RF-enriched fractions from four AxD cases (#6-#9) were analyzed by SDS-PAGE and in-gel staining (A), followed by immunoblotting using anti-ubiquitin (B, lanes 1-4) and anti-GFAP-δ (B, lanes 5-8) antibodies. Immunoblot with merged signals was shown (B, lanes 9-12), with ubiquitin signals indicated by Ub_1-3_ and Ub_n_. Molecular weight markers (in kDa) were indicated on the left. (C and D) RF-enriched fractions were prepared from hippocampus (Hip) and brain stem (BS) of WT and R237H rat (C). The same fractions were prepared from indicated brain regions of WT and Tg mice (D). Protein samples were analyzed by SDS-PAGE, followed by in-gel staining to assist comparison of equal protein loading. Molecular weight markers (in kDa) were indicated on the left.


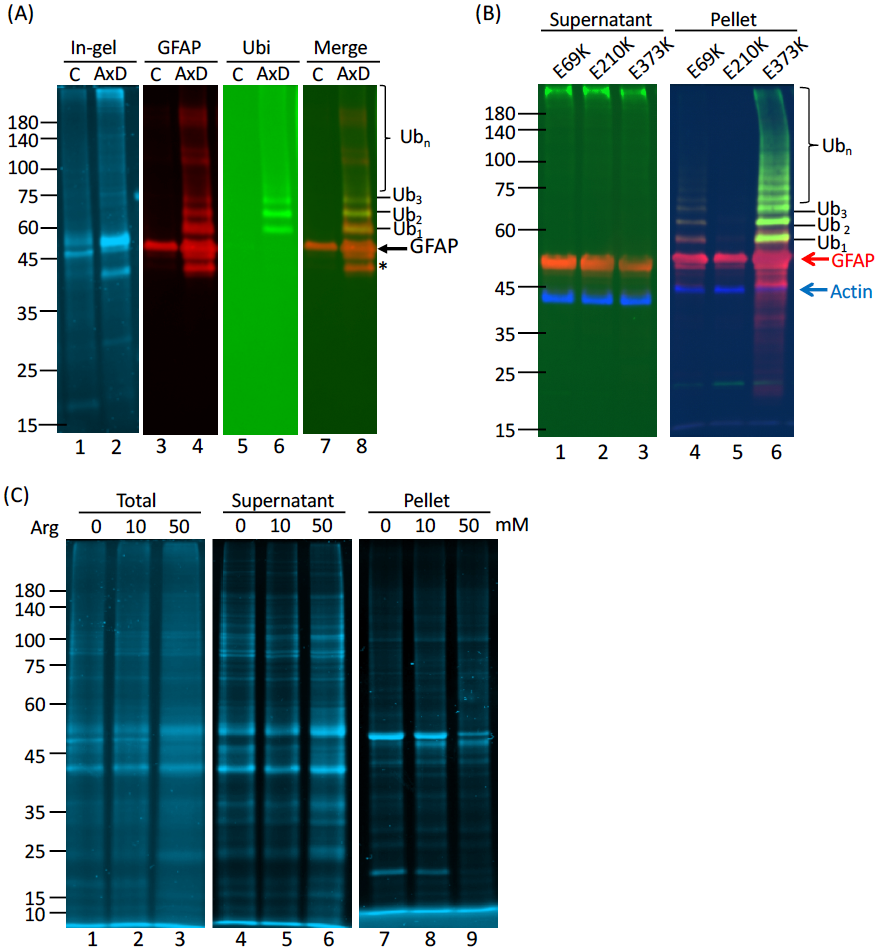


**SFig. 6.** (A) Rosenthal fiber-enrich fractions prepared from non-AxD control (lanes 1, 3, 5, and 5) and a patient with AxD carrying the E373K GFAP mutation (lanes 2, 4, 6, and 8) were analyzed SDS-PAGE, followed by either in-gel staining (lanes 1 and 2) or immunoblotting using anti-GFAP (lanes 3 and 4) and anti-ubiquitin (lanes 5 and 6) antibodies. An immunoblot with merged signals was shown (lanes 7 and 8). (B) Primary astrocytes derived from GFAP knockout (KO) rats were transduced with indicated GFAP expression constructs. At 72 h after transduction, cells were extracted and the supernatant (lanes 1-3) and pellet (lanes 4-6) fractions were analyzed by immunoblotting using anti-ubiquitin (green channel) and anti-GFAP (red channel) antibodies. Equal loading for the supernatant and pellet fractions were verified using an anti-actin antibody (blue channel). (C) SW13 (Vim-) cells were transduced with E373K GFAP in the presence of 10 mM or 50 mM arginine. At 48 h after transduction, cells were extracted and the total lysates (lanes 1-3), supernatant (lanes 4-6), and pellet (lanes 7-9) fractions were analyzed by SDS-PAGE followed by in-gel staining to assist comparison of equal protein loading of each lane. Molecular weight markers (in kDa) were indicated on the left.
